# Glioblastoma Treatment: State-of-the-Art and Future Perspectives

**DOI:** 10.3390/ijms23137207

**Published:** 2022-06-29

**Authors:** Alejandro Rodríguez-Camacho, José Guillermo Flores-Vázquez, Júlia Moscardini-Martelli, Jorge Alejandro Torres-Ríos, Alejandro Olmos-Guzmán, Cindy Sharon Ortiz-Arce, Dharely Raquel Cid-Sánchez, Samuel Rosales Pérez, Monsserrat Del Sagrario Macías-González, Laura Crystell Hernández-Sánchez, Juan Carlos Heredia-Gutiérrez, Gabriel Alejandro Contreras-Palafox, José de Jesús Emilio Suárez-Campos, Miguel Ángel Celis-López, Guillermo Axayacalt Gutiérrez-Aceves, Sergio Moreno-Jiménez

**Affiliations:** 1 Radioneurosurgery Unit, National Institute of Neurology and Neurosurgery Manuel Velasco Suárez, Mexico City 14269, Mexico; drjano@live.com.mx (A.R.-C.); juliamoscardini@outlook.com (J.M.-M.); j.alejandro.torres.rios@gmail.com (J.A.T.-R.); dralaurahernandez33@gmail.com (L.C.H.-S.); jcheredia97@hotmail.com (J.C.H.-G.); mdconfox@gmail.com (G.A.C.-P.); joseemilio53@gmail.com (J.d.J.E.S.-C.); ma.celis@innn.edu.mx (M.Á.C.-L.); neuroaxa@gmail.com (G.A.G.-A.); smoreno@innn.edu.mx (S.M.-J.); 2 Hospital de Especialidades No.1 Centro Médico Nacional del Bajío, León 37680, Mexico; alex_olmos13@hotmail.com (A.O.-G.); drasharonortiz@gmail.com (C.S.O.-A.); 3 Centro Médico Nacional Siglo XXI, Instituto Mexicano del Seguro Social, Mexico City 06720, Mexico; dhare227@gmail.com (D.R.C.-S.); samuel.rosales@me.com (S.R.P.); 4 Hospital General Regional #20, Instituto Mexicano del Seguro Social, Tijuana 22115, Mexico; charo.macias18@gmail.com; 5 American British Cowdray Medical Center, Cancer Center, Mexico City 01120, Mexico

**Keywords:** glioblastoma, temozolomide, radiotherapy, immunotherapy, target therapy, neurosurgery

## Abstract

(1) Background: Glioblastoma is the most frequent and lethal primary tumor of the central nervous system. Through many years, research has brought various advances in glioblastoma treatment. At this time, glioblastoma management is based on maximal safe surgical resection, radiotherapy, and chemotherapy with temozolomide. Recently, bevacizumab has been added to the treatment arsenal for the recurrent scenario. Nevertheless, patients with glioblastoma still have a poor prognosis. Therefore, many efforts are being made in different clinical research areas to find a new alternative to improve overall survival, free-progression survival, and life quality in glioblastoma patients. (2) Methods: Our objective is to recap the actual state-of-the-art in glioblastoma treatment, resume the actual research and future perspectives on immunotherapy, as well as the new synthetic molecules and natural compounds that represent potential future therapies at preclinical stages. (3) Conclusions: Despite the great efforts in therapeutic research, glioblastoma management has suffered minimal changes, and the prognosis remains poor. Combined therapeutic strategies and delivery methods, including immunotherapy, synthetic molecules, natural compounds, and glioblastoma stem cell inhibition, may potentiate the standard of care therapy and represent the next step in glioblastoma management research.

## 1. Introduction

Glioblastoma (GB) is one of the most lethal malignancies in the human body and the most common primary brain tumor, representing a significant challenge in neuro-oncology. Unfortunately, since Roger Stupp et al. described the current standard treatment more than fifteen years ago, the prognosis remains poor. The long-term survival has only been slightly modified, despite incessant efforts in basic, translational and clinical research [1,2].

The Stupp protocol includes maximally safe surgical resection, followed by involved-field radiotherapy (RT) (60 Gy in 2.0 Gy fractions on weekdays) over a six-week period, (42 days) with daily concomitant temozolomide (TMZ) (75 mg/m^2^) chemotherapy, followed by six cycles of adjuvant TMZ maintenance (150–200 mg/m^2^), administered for five days every 28 days [1,3].

Even though therapy is always followed by tumor recurrence and progression, recent advances in multimodality therapy have improved the median survival to approximately 15 months (14–21 months), the progression-free survival (PFS) to 10 + 1 months before recurrence, the one-year survival rate to 41.4%, and a five-year survival rate to 6.8% [1]. On one side, there are well-established negative prognostic factors, such as advanced age, poor performance status, and incomplete extent of resection. On the other side, molecular features, such as isocitrate dehydrogenase 1 (IDH-1), IDH-2 mutation, and MGMT methylation confer a favorable prognosis [2,3].

Multiple treatment options for recurrences have evolved in the previous decade, including systemic therapy, such as bevacizumab (BEV), nitrosoureas, immunotherapy, such as vaccine therapy, checkpoint inhibitors, and CAR T cell therapy, or oncolytic viruses, among others. In some cases, first-line approaches can be employed again in recurrences. There is not a well-defined standard of care for tumor recurrences, due to the lack of evidence to improve overall survival (OS) [3,4].

This article aimed to review the state-of-the-art and current guidelines in the treatment of GB, as well as future perspectives in the management of the neoplasm, including the most recent therapeutic approaches in immunotherapy, new synthetic molecules, and natural compounds.

## 2. Historical Perspective

The neuropathologists Percival Bailey and Harvey Cushing were responsible for the modern classification of gliomas in 1926, nearly 100 years after gliomas were first described and more than 50 years after Virchow proposed the first classification. Due to the multiform appearance of cells within the same tissue samples, Bailey and Cushing named the most clinically malignant and histologically unusual form of glioma Spongioblastoma Multiforme [5,6].

Due to the unusual and polymorphic monstrous cells, which exhibit no resemblance to healthy glial or even other glioma cells, they were convinced that this tumor type had a different biological genesis than other gliomas. Although astrocytoma arises from astrocytic glia and their neoplastic cells bear some similarities, spongioblastoma multiforme was considered a different type of tumor, according to their classification. The word spongioblastoma was eventually ruled out in favor of glioblastoma, establishing the common origin of astrocytoma and glioblastoma multiforme [5,6]. 

Since the description of GB, many treatment approaches have been tried to fight this cancer. As a result, a lot of knowledge and advances have been achieved; nevertheless, patients with glioblastoma do not yet have a favorable prognosis. An important landmark in the timeline of GB treatment occurred in 2005 when Stupp et al. published the results from their GB treatment protocol, based on surgical resection, radiotherapy, and temozolomide. Nowadays, it is still recognized as the gold standard for this type of tumor. 

Before 2005, surgical resection was the gold standard for patients diagnosed with glioblastoma, with a median survival time of 13 months for those with 98% of tumor resection [7]. Then, radiotherapy was added to treatment guidelines, showing a discreet benefit for patients with primary and recurrent tumors [8]. Last, chemotherapy (CT) was introduced to the GB treatment scheme with temozolomide, which was first used in cycles for recurrent tumors [9]. All these advances provide a slightly better prognosis for patients with GB.

## 3. Glioblastoma Pathophysiology

Mutations in IDH1 and IDH2 are among the most well-studied metabolic disturbances in gliomas, including GB. IDH1 is a citric acid (Krebs) cycle enzyme that converts isocitrate to a-ketoglutarate (a-KG) and is essential to produce adenosine triphosphate (ATP) during cellular energy production. During the Krebs cycle, isocitrate, produced by the isomerization of citrate, is oxidized and decarboxylated. The IDH enzyme surrounds the isocitrate in its active site by amino acids, such as arginine, tyrosine, and aspartic acid. During the first stage of the reaction, carbon #2 of the isocitrate is oxidized to form oxalosuccinate. The alcohol group on this carbon is deprotonated, electrons flow to the carbon forming a ketone group and a hydride ion is removed using NAD+/NADP+ as an electron-accepting cofactor. Then, in the second stage, the oxalosuccinate is decarboxylated. A nearby tyrosine residue deprotonates oxygen from the carboxyl group, and electrons flow to carbon #2. Carbon dioxide leaves the beta carbon of the isocitrate, electrons flow to the oxygen of the ketone group, and the latter becomes negatively charged. Finally, a double bond is formed between the alpha and beta carbons. In the third stage, the double bond between carbon #2 and #3 is saturated. A lysine residue deprotonates the oxygen of the alpha carbon regenerating the ketone bond and forming a single bond between the alpha and beta carbons by taking a proton from a nearby tyrosine residue [10,11,12].

Gliomas are known to have recurrent hotspot missense mutations in IDH1 and IDH2. IDH1 (R132) and IDH2 (R132) both have mutations at a single amino acid residue (R140). Tumors have only one mutant copy of each gene. According to groundbreaking investigations, the tumor-derived IDH mutations are neo-morphic, meaning they gain new enzymatic activity and can convert -KG to (R)-2-hydroxyglutarate (2HG). IDH1 mutations produce a distinct metabolite, 2-hydroxyglutarate (2HG), promoting a hypermethylation phenotype in gliomas [10,11]. The mutant variant of IDH1 has been found to interact with the IDH-wildtype enzyme, reducing its activity. Gain-of-function mutations in IDH1 result in the synthesis of the oncometabolite 2HG from a-KG. As a result, 2HG levels are higher in IDH1 or IDH2 mutant gliomas than in IDH wild-type tumors [12,13].

In gliomas, the hypoxia-inducible factor 1-alpha (HIF-1a) is a significant pro-angiogenic and pro-glycolysis transcription factor that is increased in IDH1 mutant GB cells. This transcription factor targets GLUT1, VEGF, and PDK1 genes. Prolyl hydroxylase (PHD) enzymes, which inhibit HIF-1a, are inhibited by IDH1 mutations and 2HG generation [12,14].

The occurrence of a CpG island hyper-methylator phenotype (CIMP) is a second change linked to IDH1 mutations, which displays distinct CpG island methylation at a more significant number of locations than non-IDH1 mutants and primary GB, according to a genome-wide methylation profile investigation in gliomas. In addition, the induction of mutant IDH1 into human astrocytes produces functional changes, and, in particular, histone markers by impairing histone demethylation and inducing DNA hypermethylation. As a result, in gliomas, the IDH1 mutation has an important role in hypermethylation [12,15].

A sequence of human tumors for IDH1 or IDH2 mutations, or monoclonal antibodies against the most common IDH1 mutation (R132H), allows for immunohistochemical analysis of low-grade gliomas and GBMs. Now, it is possible to use MRI spectrometry to detect the IDH mutant’s oncometabolite, 2HG, which may allow a noninvasive classification of the grade and subtype of glioma. This technique may be used to evaluate responses of potential treatments against IDH mutant tumors [16].

The O6-methylguanine-DNA methyltransferase (MGMT) gene encodes for a DNA repair protein that removes alkyl groups from the O6 position of guanine, which is a key location for DNA alkylation. Chemotherapy-induced alkylation causes cytotoxicity and apoptosis at this location. Tumor cells that overexpress the MGMT repair protein could be capable of blocking the therapeutic effects of alkylating drugs. In over 40% of primary glioblastomas, and over 70% of secondary glioblastomas, MGMT is epigenetically inactivated by hypermethylation of the 5’-CpG island. CpG islands are genomic areas with a higher than usual frequency of CG dinucleotides (CpG sites), which are involved in gene transcription modulation [17].

CpG islands, such as the one linked to the MGMT gene, frequently span the transcription start site of genes and contain essential transcription factor binding sites. Aberrant methylation of CpG islands can cause gene transcription to be disrupted, resulting in reduced, or even complete loss of, gene product expression. However, the methylation patterns of the MGMT promoter in malignant gliomas differ widely, and it is unclear which exact CpG sites or combinations of CpG sites must be methylated to silence the gene and benefit from alkylating drug therapy [17].

The exact origin of a GB is rarely identified, but it is suggested to derive from neural stem cells (NSCs) or glial precursor cells, which have the ability to infiltrate brain tissue and cause endothelial necrosis, creating the typical histopathological inflammatory pattern. The aggressive behavior of GB is likely determined by a small subpopulation of cancer cells named glioblastoma stem cells (GSCs), which have pluripotential and self-renewal capacity. These characteristics protect GSCs from chemotherapy- and radiotherapy-induced damage. Targeting stem cells or inducing differentiation are innovative therapeutic strategies covered by new synthetic molecules and some natural compounds described later. Alternative drug delivery systems through stem cell mechanisms are already being tested in several preclinical trials [18,19].

## 4. State-of-the-Art: Surgery, Tumor Treating Fields, Radiotherapy, Chemotherapy and Bevacizumab

### 4.1. Safe Maximum Resection

Many years before the Stupp et al. trial was published in 2005, maximal safe resection surgery was the initial technique in the gold standard of treatment [1]. The surgery’s primary treatment goal is to achieve a gross total resection (GTR) as safely as feasible without risking the patient’s functional state. Tumor volume reduction, histological diagnosis, and tumor genotyping are all possible with the surgical approach, all of which are essential factors in selecting the following stages in treatment. A stereotactic or open biopsy is advised if surgical resection is not an option [2,20,21].

Full resection involves removing the entire contrast-enhancing tumor in the T1 gadolinium weighted image. Full resection has been associated with a higher chance of survival and no progression than partial resection or biopsy. Several surgical tools have been developed to assist in achieving a maximal resection of the tumoral tissue while trying to avoid, as much as possible, the neurological deficits related to the procedure. Such tools include surgical navigation systems with functional MRI (fMRI), functional monitoring, and fluorescence-based visualization of tumor tissue with 5-aminolevulinic acid (5-ALA) or fluorescein. In addition, when a tumor involves eloquent areas, functional tools such as brain mapping in awake patients, evoked potentials, or electromyography have shown beneficial results in long-term neurological functional outcomes [21,22,23].

ALA is a body-produced metabolite in the biosynthesis pathway that is given as a 20 mg/kg body-weight oral solution. This molecule is rapidly absorbed and eliminated from plasma within 2 h of treatment, due to its small size. After 6 to 8 h, a peak fluorescence can be expected, with fluorescence being evident after 3 h. Gliomas selectively take up ALA and convert it to protoporphyrin IX (PPIX) via enzymes in this pathway. Many investigations have shown that ALA-induced PPIX has a high selectivity, although normal brain tissue does not develop PPIX in response to ALA exposure [24,25]. All of the main current surgical microscopes have adjuncts that can visualize PPIX. Filtered xenon light with a wavelength of 375 to 440 nm and an emission filter that allows viewing red fluorescence with a peak at 635 and 704 nm are required to visualize PPIX fluorescence. The filters are also designed to let some of the excitation light and green autofluorescence emitted by the tissue to pass through, allowing background discrimination and fluorescence surgery over more extended periods of time [24,25,26,27].

Fluorescein was the first agent to be utilized intraoperatively for better tumor detection and identification. It is administered intravenously in doses ranging from 3 mg to 20 mg/kg during induction of anesthesia, prior to dural opening, or acutely, during resection, using either dedicated microscopes or microscopes without any specific adjuncts for fluorescence visualization. Due to the extra time between injection and resection, fluorescein is eliminated from the dura and venous system, and is only retained in locations where the blood-brain barrier has been damaged, allowing for tumor delimitation [24,26,28].

It is important to remember that gliomas are not cured by surgery alone. Nowadays, even though the extent of resection is a prognostic factor, and efforts at obtaining complete resections are always justified, the priority is to prevent neurological deficits caused by surgery. Neurological deficits arising from surgery cause reduced independence and quality of life, which lead to increased complications that may even impede the following steps in the standard management, such as radiotherapy or chemotherapy, which have more impact in the final overcome than the extent of resection [22,23,26].

All patients have a different clinical presentation of their disease, and some of them may have some negative prognostic factors that surgical procedures can modify. For example, the initial prognosis for multicentric lesions or multifocal tumors is poor, but surgical resection management improves it. Another significant poor prognostic factor is when a GTR is not accomplished and significant post-surgical residual tumor volumes remain [3,23].

Recent research suggests that the tumor’s biological features may influence its resectability. Some uncontrolled retrospective studies observed that the rate of GTR was higher in IDH-mutant tumors than in IDH-wildtype tumors. Less malignant brain tumors may be more resectable than tumors with more aggressive biological characteristics. This consideration does not discourage the efforts to achieve gross total resection when feasible. If possible, GTR is advised in recurrent cases with a time interval of >6 months since the first surgery, especially in younger patients with a good clinical state [2,27,29].

#### Awake Craniotomy

Awake craniotomy (AC) with intraoperative cortical electrodes for motor and speech monitoring has obtained outstanding results. It is the current gold standard technique for diffuse brain tumor resections, due to its capacity to identify and preserve cortical and subcortical functional areas. The main aim of AC is to preserve motor and speech functions and achieve a complete resection of the tumor [22].

For a successful outcome, suitable patients must be chosen. Uncontrolled chronic cough, hemiplegia with motor function <2 on the Daniel’s scale, severe dysphasia, and big tumors with mass effect resulting in >2 cm of midline shift are all absolute contraindications for awake craniotomy [27]. However, individualization of the patient is essential, considering that adaptation is possible in some instances [22,29]

During AC, different anesthetic approaches are employed, including conscious sedation (CS) and the asleep-awake-asleep procedure (AAA). Conscious sedation entails supplementary oxygen, spontaneous ventilation, and modest doses of sedative medications. Dexmedetomidine has been demonstrated to decrease the number of sedatives and opioids required. It has strong effectiveness and a strong safety record [23,27,30,31].

The AAA technique uses general anesthesia before and after cortical mapping and functional testing. Drug infusion is interrupted 15 min before the functional testing, and ventilatory support is removed when the patient obeys commands [22]. When the neurological examination is completed, anesthesia is induced, and ventilatory support is restarted. The primary goals of this technique are to maintain the pre-awake state, shield the patient from discomfort, reduce brain swelling through hyperventilation, and restrict patient movement during operation [2,22,30,31].

Intraoperative magnetic resonance imaging (I-MRI) is a technique that has been used in addition to mapping during awake craniotomy. This tool provides real-time intraoperative MRI images that detect the tumor and its remnants, which allows better precision, enabling consideration of the changes in the brain anatomy during surgery. The combination of both techniques enables maximum resection while minimizing neurological deterioration [22,31].

### 4.2. Radiotherapy

Radiotherapy (RT) has been a cornerstone in the treatment of GB for more than fifteen years. The main goal of RT is to improve local control without inducing neurotoxicity. Current guidelines recommend 60 Gy administered in 2.0 Gy fractions on weekdays for six weeks for first-time treated GB (from Monday to Friday), starting 3–5 weeks after surgery. RT usually starts 3–5 weeks after surgery. When CT/RT is received >5 weeks after surgery, a reduction of 3 months in PFS has been observed in a retrospective study. The inter-lapse between surgery and RT/CT is inversely related to PFS and OS. However, tumor recurrence and progression virtually always occur after treatment [2,21,32].

A planned goal volume should include the gross tumor volume (GTV = area of surgical bed + residual tumor area in T1WI, T2WI/FLAIR sequences) and a clinical target volume that involves a 1–2 cm margin to account for microscopic invasion. Also, a 0.3–0.5 cm margin is added, considering the uncertainties that may coexist. The administration dosages should be 50–60 Gy in 1.8–2 Gy daily portions over six weeks. Other radiotherapy doses, schemes, and ionizing radiation techniques have been tested for primary treated GB without conclusive results [32].

According to the Karnofsky performance scale (KPS) index, radiotherapy doses can be adjusted. When compared to supportive treatment alone, a dose of 50 Gy in 1.8 Gy fractions provided an OS benefit for elderly (>70 years) patients with a good functional status (KPS > 70) (29.1 weeks vs. 16.9 weeks). A hypo-fractionated regimen of 40 Gy in 15 fractions of 2.67 Gy over three weeks has shown equivalent survival outcomes to standard dosages in patients with poor functional conditions (KPS < 70) [2,32].

Adaptive RT is gaining popularity in the treatment of GB. It consists of the application of RT and a subsequent evaluation of changes in tumor size and form by sequential CT/MRI scans. The dosages are modified based on the new circumstance, usually lowering complications and enhancing the long-term quality of life by minimizing radiation to adjacent normal tissues. Studies have shown adaptive RT to improve irradiation efficacy in the target volume and lower the dosage received by organs at risk while also increasing local control, OS, and PFS. However, further research is needed to determine the benefits of adaptive RT [2,3,23,32].

Recently, studies evaluating the combination of hypo-fractionated RT and concurrent TMZ showed a better OS (9.3 months) than radiation alone (7.6 months) (HR, 0.67; 95 percent CI, 0.56–0.80 [*p* < 0.001]) with no differences in quality of life. However, these trials did not compare the groups to standard-of-care (SOC) RT + TMZ. Further studies comparing hypo-fractionated RT + SOC should be conducted [2,32,33].

For recurrences, radiotherapy has been studied as a potential treatment alternative, particularly for younger patients with good performance status. However, several radio-resistance mechanisms, developed on the first RT course, have put the supposed benefits of re-irradiation in question. For recurrences, various ionizing radiation treatments have been investigated, including highly conformal radiation techniques, such as intensity-modulated RT, proton or heavy ion irradiation, stereotactic radiotherapy (SRT), radiosurgery (SRS), and hypo- and hyper-fractioned regimens. Nevertheless, more randomized control trials (RCTs) are required to determine these approaches’ tolerability, safety, and efficacy compared to standard radiotherapy [4,33]. Recently, new synthetic molecules and natural compounds have demonstrated radiosensitizer properties in pre-clinical trials. Results are discussed later in the text.

In GB recurrence, the efficacy and safety of SRS and SRT have been investigated. In a trial using SRS, patients who received a median dose of 24 Gy in four fractions had a median OS of 14.6 months after treatment, with no toxicities registered. Safety and outcome improvement has been reported in retrospective evidence about SRS and short courses of hypo-fractionated SRT in GB recurrences. An ongoing prospective phase II study (NCT04197492) is investigating the value of hypo-fractionated SRS in recurrent HGG [2,32,33].

About the efficacy of gamma knife radiosurgery (GKRS) in high-grade glioma (HHG) recurrences, a study observed a median OS of 13 months and a survival rate of 51.4% at 1-year, 10% at 2-years, and 2.9% at 5-years, which can represent a different area of interest [32].

A combination of RT and systemic treatments, such as bevacizumab, has already been investigated in GB recurrence. After delivering a combined therapy of BEV+ RT/TMZ to patients in phase III studies, the results showed only a PFS improvement with no changes in OS. Recent research has found similar findings in PFS with a tolerable toxicity profile [32]. Kulinich, on the other hand, conducted a comprehensive evaluation of data from patients with recurrent GB who had been treated with SOC therapy and then retreated with the BEV+ RT combination as a second therapy. After multivariate analysis, they discovered a slight improvement in OS but no meaningful advantage in PFS. Furthermore, the data revealed that patients who took BEV had a much lower rate of radio-necrosis [2,32,33].

The combined therapy of BEV + RT is an optimistic regimen for the GB recurrence instance, which has shown acceptable safety profiles, improvement in OS, and potential reduction of radio-necrosis. Nevertheless, the inconsistency between trial results exhibits the need for further investigation that includes analysis of the possible patient and tumor characteristics involved in the outcome [2,32,34].

Several ionizing radiation treatments and regimens have been examined, particularly in the GB recurrence scenario. Nonetheless, current guidelines only agree on SOC conventional radiotherapy for first-time GB patients. Although the significance of re-irradiation in recurrent GB is unclear, research into combined BEV+ RT, SRS, SRT, and GKRS seems promising [2,32,34].

Aside from the well-known effects of radiation on tumor cells, DNA alkylation, endothelial damage, and the creation of free radicals, the effects on cell membrane proteins are crucial. Proteoglycans (PGs), for example, are implicated in initial glioblastoma development and contribute to cancer stem cell (CSC) treatment resistance and GBM recurrence development. Radiotherapy reduces brevican and neurocan concentrations in cerebrospinal fluid at 12-months following irradiation [35]. Hyaluronic acid (HA) is a PG component of any tissue but plays an especially critical role in the brain. It comprises a significant component of intercellular space, is involved in GB pathogenesis, and is damaged by radiotherapy. RT causes a considerable rise in HA content. In addition, HA interaction with the CD44 receptor induces a mesenchymal shift in GBM cells. An increase in HA content on the tumor tissue affects the microenvironment, providing pro-invasive extracellular signaling [35].

Other essential effects induced by RT are early metabolic responses in the tumor and the adjacent tissue during the first week of RT. Glutamate is a non-essential amino acid and a primary excitatory neurotransmitter. It has been linked to the invasive process and a high frequency of seizures in patients with HGG. Glioma cells have been shown to release a high glutamate concentration, causing widespread excitotoxic death in normal neurons, whereas normal astrocytes remove glutamate from the extracellular space. This process promotes tumor growth and invasiveness. Glutamate levels rise after radiation and have been proposed as a marker for ischemia and traumatic brain injury. The radiation-induced glutamate increase could be due to a release from the tumor or astrocytic cells injured by radiation [36].

Myo-inositol is a crucial intracellular and second messenger molecule and is involved in signaling, and is abundant in brain adjacent tissue (BAT). Myo-inositol promotes the generation of phosphatidylinositol, which is then used to produce diacylglycerol and inositol 1,4,5-trisphosphate (IP3). Protein kinase C and a cascade of proteolytic enzymes, including matrix metalloproteases, are activated as a result of the diacylglycerol, which plays an essential role in tumor invasion. In both the tumor and the BAT, there is a rise in inositol. A variety of signaling and secondary messenger molecules are based on inositol and Myo-inositol. Ca^2+^ is released when the IP3 receptor is stimulated by IP3, rendering the cells more sensitive to apoptotic triggers. The inositol rise is caused by a decrease in the synthesis of IP3, which leads to decreased sensitivity to apoptotic stimuli, enhancing glioma cell resistance to radiation-induced apoptosis [36].

S-methyl-L-cysteine levels are more significant in tumors than in BAT. S-methyl-L-cysteine is the end product of a methylated-DNA-cysteine S-methyltransferase mediated demethylation process of DNA containing methylguanine. S-methyl-Lcysteine levels in tumor tissue drop after RT, suggesting that the treatment interferes with the methylated-DNA-cysteine S-methyltransferase-mediated demethylation process [36].

### 4.3. Chemotherapy: Temozolomide (TMZ)

Temozolomide, an oral DNA alkylating drug that penetrates the blood-brain barrier, is the current first-line, and most used, systemic therapy for GB. In its passage into the cytoplasm, TMZ undergoes spontaneous hydrolysis to form monomethyl triazene 5-(3-methyltriazene-1-yl)-imidazole-4-carboxamide (MTIC). This compound is then hydrolyzed to form the final cation methyldiazonium. This active cation adds a methyl group to purines and pyrimidines in DNA, specifically in the N^7^ position of guanine (70%), guanine rich sites, and to a lesser extent in N^3^ adenine (9%), and O^6^ guanine residues (6%). These methylation modifications result in damage to cells, apoptosis, and cell cycle arrest at the G2/M phase [37]. During concurrent RT, the daily optimum dose is 75 mg/m^2^ for a six-week period (42 days), followed by six cycles of maintenance of 150–200 mg/m2 for five days every 28 days. In patients with poor performance status (KPS < 70), it is suggested to administer TMZ alone at 150–200 mg/m^2^ for five days every 28 days after surgery. For newly diagnosed GB, there is no evidence of benefit from different TMZ doses or treatment strategies [2,4].

Patients that will benefit from the treatment are those whose tumors have aberrant CpG methylation of the promoter area of the DNA repair enzyme MGMT gene. This methylation limits the transcription of an enzyme involved in DNA reparation following the genotoxic effects of alkylating chemotherapy. Approximately 55% of GB patients are resistant to TMZ because of their MGMT DNA repair system. MGMT transfers the methyl group from guanine, thereby repairing damaged DNA and counteracting the cytotoxic effects of TMZ on tumor cells [37]. Studies have indicated that patients with MGMT methylated tumors have a better prognosis, with a median 2-year survival rate of 46%, implying that TMZ is exclusively active in this kind of GB, with only a minor effect on MGMT unmethylated tumors [20,21].

Unfortunately, evidence indicates an evolution to recurrence in almost every patient at six months within the standard treatment. In this instance, no standard treatment is established, but the most commonly employed systemic therapies are alkylating agents like TMZ rechallenge or nitrosoureas, such as lomustine and carmustine. The MGMT methylation status plays the same role as in primary management. Low-grade evidence studies on individual chemotherapy indicate minimal changes in OS and greater toxicity with other regimens [4,23].

Myelosuppression, particularly thrombocytopenia, neutropenia, nausea, and hepatic damage, are the most common side effects of alkylating drug treatment. Even though adverse effects are typical during the adjuvant phase, the hepatic function should be examined regularly in patients receiving TMZ [2,3]. The use of nanotechnology to increase chemotherapy administration to the CNS via the blood-brain barrier (BBB) is a promising alternative that is still in research [20,21].

Nitrosoureas, such as lomustine and nomustine, are oral alkylating drugs that have been used to treat GB. Their mechanisms consist in alkylate DNA and RNA, as well as cross-link DNA, acting both in and out of the cell cycle. The production of O6 -chloroethylguanine, which can be reversed by MGMT, is one of the most important lesions generated by lomustine [38]. By carboxylation of amino acids, such as lysine or arginine, lomustine may impede enzymatic processes, leading to cell death through TRC8-mediated degradation targeting heme oxygenase-1 [39]. However, the clinical relevance of this action is uncertain. As a result of its lipo-solubility, it easily crosses the BBB, making it a viable candidate for treating intrinsic brain cancers [38,40].

Lomustine has recently been tested in combination with TMZ to observe a possible booster effect of TMZ on lomustine efficacy to deplete MGMT. In patients with MGMT promoter methylation GB, this treatment proved to have an elevated survival outcome. Overall, there was longer survival for the temozolomide-lomustine combination over SOC in a randomized phase III clinical trial (CeTeG). This finding shows that different alkylating agents may have actual synergistic properties that merit further investigation [38,41].

For now, TMZ remains the first-line treatment for primary and recurrent GB management, particularly for MGMT promoter-methylated tumors. Recurrences can be treated with other alkylating drugs, such as nitrosoureas like lomustine. In any case, combined chemotherapy regimens, or increased TMZ doses, have limited advantages and higher toxicity.

### 4.4. Tumor-Treating Fields (TTFs)

Tumor-treating fields are a newly approved physical treatment that uses transducer arrays applied directly to the scalp to give low-intensity (1–3 V/cm), intermediate-frequency (200 kHz) alternating electric fields to treat newly diagnosed or recurring GB. TTFs generate selective toxicity in quickly dividing cells by causing neuronal depolarization and disrupting microtubule formation during mitosis. Since 2015, the FDA has approved this treatment technique as an adjunct therapy for recurrent gliomas [2,42].

A phase III trial conducted by Stupp et al. reported a PFS improvement of 6.7 months for the maintenance TMZ + TTF group versus 4.0 months for the maintenance TMZ-alone group (HR, 0.63; 95 percent CI, 0.53–0.76 [*p* < 0.001]) and an OS benefit of 20.9 months vs 16.0 months for the maintenance TMZ-alone group (HR, 0.63; 95 percent CI, 0.53–0.76 [*p* < 0.001] (HR, 0.65; 95% CI, 0.53–0.76 [*p* < 0.001]) [2,42].

TTFs have been tested in phase II and III trials in both first-time treated and recurring GB patients. PFS, OS outcomes, and objective responses improved as a result of the study. However, disagreements over study design, execution, and data interpretation have raised questions about the current evidence. Furthermore, the cost of completing the therapy is a further impediment to TTFs being used regularly [2,4,33].

There is plenty of evidence generated in the last years about the benefits of TTFs in GB. This therapy currently represents a potential alternative for the management of newly diagnosed and recurrent GB patients. However, despite the advances, the debate remains open about the limitations of this novel technique, the costs of which, together with its poor accessibility, have limited its regular application in most neuro-oncologic centers. 

### 4.5. Bevacizumab

GBs are highly vascularized tumors characterized by overexpression of vascular endothelial growth factor (VEGF), a key regulator of tumor-associated angiogenesis. VEGF is a major target recently explored in most therapeutic trials. Bevacizumab (BEV) is a humanized monoclonal antibody against VEGF that has proven a prolonged PFS (3–4 months) but not OS benefit at several phase II and III clinical trials in newly diagnosed and recurrent GB [20,33,43].

Particularly in the recurrence setting, BEV presented response rates of approximately 30% in uncontrolled phase II trials [43]. About co-adjuvant chemotherapy with BEV, a randomized phase III trial tested the combination of BEV + lomustine versus lomustine alone, and results showed an improvement in PFS without OS changes in the combination group [44,45,46].

Another common combination for recurrences is BEV + re-irradiation. Two phase III trials found that BEV + RT-TMZ combination therapy increased PFS but not OS, as in practically every clinical trial. On the other hand, re-irradiation plus BEV was studied by Kulinich et al. The results showed a significant OS improvement but no significant PFS benefit [46]. Remarkably, patients who had previously been irradiated and were given BEV presented a reduced incidence of radio-necrosis. These findings show that the efficacy of RT with BEV is highly variable. As a result, the usefulness of this combination is still up for debate, and more randomized studies will be needed to determine the benefit [16,19,20,21]. However, neither combination therapy had demonstrated OS benefit, and the mentioned regimens are only recommended after failure of bevacizumab alone [2,32].

Based on high radiological response rates and the optimistic PFS outcomes described, bevacizumab achieved FDA approval for recurrent GB in many parts of the world, such as the USA, Canada, and Switzerland, but its effects on tumor biology and growth dynamics remain controversial [20,32]. Its failure, in clinical trials, to demonstrate an OS benefit has either stopped approval of BEV therapy for newly diagnosed GB management or has slowed down the approval process, as has happened in many regions, such as the European Union, where it remains not approved, even in the recurrence setting [33].

A relevant aspect of BEV is how it affects patients’ cognitive abilities, symptoms, and quality of life. There is strong evidence that patients using BEV had worse scores on objective tests of neurocognitive function and reported cognitive function compared to placebo, implying either undiscovered tumor progression or BEV-related neurotoxicity. Furthermore, among patients who did not have tumor development on imaging investigations, those initially treated with BEV reported a worsening in the severity of their symptoms, as measured by both patient-reported outcomes and symptom-related interference with daily activities [47]. On the other hand, some research suggests that BEV patients have a considerably longer deterioration-free life than placebo patients after a year of treatment. When examining cognitive functioning, emotional functioning, role functioning, weariness, visual dysfunction, weakness in both legs, hair loss, bladder control, and financial difficulties, patients in the BEV group have a considerably longer time before deterioration [43]. Thus, more data is necessary to determine the real impact of BEV on patients’ symptoms and quality of life.

As far as evidence suggests, BEV has recently been included among the main systemic treatment options for GB progression or recurrence after its approval by the FDA as a viable therapeutic alternative. This anti-angiogenic therapy has been subjected to different trials combined with novel immunological therapies presented further in the text.

### 4.6. Standard of Care

The current guidelines for newly diagnosed IDH-wildtype GB, WHO grade 4, treatment agree to indicate maximal safe resection as the first step in all patients. For younger patients aged <70 years with a good performance status (KPS > 70) the surgery must be followed by involved-field RT (60 Gy in 1.8–2.0 Gy fractions) + TMZ (75 mg/m2 daily throughout RT, including weekends) + 6 cycles of maintenance temozolomide (150–200 mg/m^2^, 5 out of 28 days) + TTFs. An alternative in MGMT promoter-unmethylated tumors is surgical resection and RT alone. For poor performance status (KPS < 70) hypo-fractionated RT (40 Gy in 15 fractions) + TMZ or TMZ alone or best supportive care (BSC) are considered. Limiting the addition of TMZ for patients with MGMT promoter-methylated GB is to be considered [1,48].

For aged patients (>70 years) with good performance status, a regimen of RT + concomitant TMZ followed by TMZ maintenance + TTFs is recommended after GTR. The use of TMZ alone in MGMT promoter-methylated tumors is an acceptable alternative. TMZ alone or BSC is suggested for patients with low-performance status [2,33]. Accelerated hyper-fractionated, hypo-fractionated, brachytherapy, radiosurgery, or stereotactic radiotherapy are not, at this time, considered superior to average radiation in OS [33,48].

The SOC for recurrences or relapses is not well established. It is usually chosen based on the first therapy used and prognostic markers, such as age, KPS, MGMT promoter methylation status, and disease development trends. A second surgery, systemic therapy, BSC, re-irradiation, or TTFs are all indicated as alternatives in this case. BEV, TMZ rechallenge, nitrosoureas, such as lomustine/carmustine, and a combination of BEV and chemotherapy are the main systemic alternatives, but their impact on OS is still unclear [23,33,34,48].

On the other hand, BEV has not been approved to treat newly diagnosed GB but could be helpful in large and highly symptomatic tumors that might not otherwise tolerate RT [4]. The immunotherapy approach continues to be studied in several clinical trials, which are mentioned below.

## 5. Future Perspectives: Immunotherapy

Recently, immunotherapy has been demonstrated to be a highly effective therapy against solid tumors, such as clear cell renal carcinoma, non-small-cell pulmonary carcinoma, and melanoma, which has become a crucial SOC [49]. However, for now, these novel cancer therapy results have not been precisely traduced to GB, a situation that can be explained by the extreme complexity of immunological function in the CNS.

As a consequence of its unique microenvironment and absence of immune reactivity, the CNS is usually referred to as an immunological sanctuary. The BBB is the first line of defense against infections, immune cells, and antibodies; therefore, the almost complete absence of antigen-presenting dendritic cells is replaced by microglia, the primary antigen-presenting cells in the CNS, resulting in an immunosuppressive environment. Lymphatic veins running through the brain’s venous sinuses have recently been discovered, and their discharge feeds to deep cervical lymph nodes [50,51]. In addition, the robust immune response against pathogens at infections, and trigger antigens in autoimmune diseases, shows certain similarities to the immunologic function in the rest of the body. Despite the particularities, recent observations about the CNS immunological function have demonstrated that it may contain the appropriate conditions for the application of cancer immunotherapy [49].

The immune evasion and resistance mechanisms of GB play a fundamental role in the tumor’s aggressive behavior and poor outcome. Its extraordinary adaptation capability to immune response represents a major challenge for immunotherapy strategies. The cellular heterogeneity within the tumor and its constant immune editing are key points in its adaptation and resistance to treatments [52,53].

In order for GB therapy to be effective, it must overcome numerous obstacles, such as accessing the CNS through BBB, mounting a reaction within an immunosuppressed tumoral microenvironment, and inducing cellular memory to prevent relapse. Immunotherapy has the potential to overcome these obstacles, so research has focused on several approaches for mounting a precise and effective immune response against tumor cells, while avoiding organ damage. Vaccine therapy, oncolytic viruses, immune checkpoint inhibitors (CPIs), and chimeric antigen receptor T (CAR T) cells therapy are among the most promising immunotherapy strategies for GB treatment [20,49,52].

### 5.1. Vaccine Therapy

When dendritic cells detect pathological changes in tissues, they migrate to lymph nodes and present antigens to T cells through major histocompatibility complex molecules I and II, activating effector T cells. However, it is difficult to modulate antigen presentation in tumors such as GB. The goal of vaccine therapy is the induction of the patient’s own adaptive immune response that stimulates immunogenicity against cancer cells [54].

**Figure 1:**
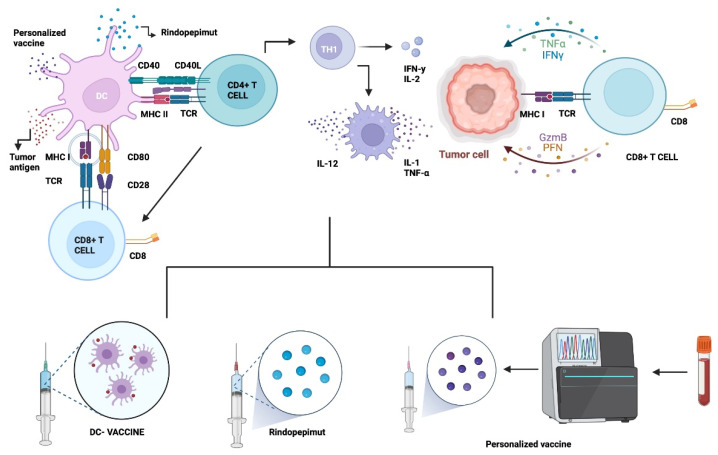
Vaccine Therapy. GB vaccines aim to generate an immune response by stimulating T cells and generating a cytotoxic response so that they attack the tumor through binding by specific receptors and MHC molecules. One of these receptors is EGFRvIII (Rindopepimut). Dendritic cell vaccines can be generated through stimulation by tumor cell lysate or peptides. Dendritic cells made with ex vivo glioma antigens migrate to lymphoid organs and activate T cells to subsequently attack the tumor. Customized vaccines are engineered through genetic engineering to the patient’s tumor-specific receptors. Abbreviations: DC: dendritic cell. MHC I: Major histocompatibility complex class I. TCR: T-cell receptor. IL-12: Interleukin-12. TNF-α: Tumor necrosis factor α CD8+ T cell: Cytotoxic T lymphocytes. Th cell: Helper T cell. CD40L: ligand CD40. Created with BioRender.com, accessed on 30 March 2022.

The mechanism starts by injecting specific tumor antigens together with an immune booster to trigger the complete response against the tumor, which in consequence will be complete or partially lysed, releasing several neoantigens or damage-associated molecular patterns (DAMPs) that may contribute to the amplification of response [49,55]. There are three main approaches in vaccine therapy against GB: tumor-specific antigens, cell-based therapies as dendritic cells, and viral vector vaccines transporting tumor antigens as mRNA. These strategies seek to minimize organ toxicity at healthy tissues (Figure 1). Other common techniques are heat shock proteins, and personalized and combined vaccines [49,50]. The most relevant current and finished clinical trials are detailed in Table 1.

#### 5.1.1. Epidermal Growth Factor Receptor Variant III (EGFRvIII)

EGFR belongs to the ErbB family of tyrosine kinase receptors implicated in GB development through several regulatory pathways, such as PI3K/AKT/mTOR and RAS/RAF/MEK. These pathways are amplified in approximately 40–60% of GB IDH-wildtype [54,56]. Some of the EGFR genes exhibit deletions that result in deficient expression of EGFRvIII, which is a tumor-specific antigen receptor expressed in 20–30% of GB [53,54]. In HGG, this is the most common gain mutation, causing a constitutive activation that is an incentive for growth and proliferation signals in tumor cells. Due to the mentioned factors, vaccines against EGFRvIII have been the first and most evaluated strategy in the field [20,49,52,57].

The first targeted vaccine research for EGFRvIII was obtained from PEP-3 peptide conjugated with limpet hemocyanin (KLH), giving it the name PEP-3-KLH peptide vaccine or CDX-111, also named Rindopepimut [54,58]. Rindopepimut was described by the FDA as a “breakthrough therapy” for GB in 2015 due to the results obtained in the ACT II study, where the therapy was well tolerated with a promising PFS and OS benefit [59]. Later, ACT III was conducted to confirm the results, obtaining adequate tolerability with a PFS of 5.5 months, and a median OS of 21.8 months [60].

This study was followed by a randomized phase III clinical trial (ACT IV) that evaluated the efficacy and safety of Rindopepimut after standard of care surgery and chemoradiation. Unfortunately, the trial was suspended, due to a lack of benefit in OS, the central point of the research. The study showed a loss of EGFRvIII expression, independently of the treatment received, which confirmed the spontaneous nature of the process in almost 25% of GBM patients. Even though patients that received Rindopepimut presented immunogenicity with a demonstrable humoral immune response. The poor results in this trial may be attributed to the selection of a single target antigen, because even though EGFRvIII is present in approximately 30% of GB tumors, only a few cells within the tumor express the antigen, and the number of targeted cells may not be significant for the whole destruction of the tumor [33,51,56,61,62]. Therefore, different treatment combinations remain to be evaluated to better assess the utility of Rindopepimut in the management of glioblastoma.

#### 5.1.2. Dendritic Cell Therapy (DC)

Physiologically, dendritic cells (DCs) are the primary antigen-presenting cells in the immune system and the first step in mounting a specific T-cell response against almost all pathogen antigens. In DC therapy vaccines, the DCs are isolated from the patient’s bloodstream and stimulated ex vivo against tumor-specific antigens. Then, the active DCs are reintroduced into the patient organism expecting the mount of a specific immune response against tumoral components [49,51,55,63]. The application of DC therapy in GB has been studied with two main vaccines at two different randomized clinical trials.

Recently developed ICT-107 is an autologous DC vaccine that targets six tumoral antigens, which include human EFGR-2 (HER2/neu), tyrosine related protein 2 (TRP-2), melanoma-associated antigen 1 (MAGE-1), glycoprotein 100 (gp100), antigen isolated from melanoma 2 (AIM-2), and interleukin-13 receptor alpha 2 (ILRαa2). The combination has been tested in a double-blind, placebo-controlled phase II clinical trial, in which one arm consisted of ICT-107 + conventional chemoradiotherapy. The results showed a median PFS slightly superior in the experimental group without benefit in OS. In the analysis, the subgroup that demonstrated immune response had improvement in PFS and OS over the patients without response [49].

On the other side, DCVax-L has been studied in a randomized, large-scale, placebo-controlled phase III clinical trial on newly diagnosed GB patients (NCT00045968), where the authors compared an experimental group with the standard of care + DCVax-L versus a control group with the standard of care + placebo. The preliminary results were reported as a single-arm because almost 90% received DCVax-L therapy at some point, no matter their original group because they were offered compassionate access to the experimental maneuver after progression. Due to this exception, PFS results are not available, but the median OS reported was 23.1 months post-surgery compared to historical control groups of other similarly designed clinical trials. There are some concerns about the study results because of the possibility of a selection bias of patients with favorable outcomes and the methodology followed to compare OS confidence intervals with the control groups of external trials [64].

Other clinical trials have been conducted using DC therapy for newly diagnosed and recurrent GB, most of them reaching phase II with a low sample of patients. However, although most of them prove safety and efficacy, they do not achieve an increase in OS when compared with the standard. Promising results have been obtained when standard treatment is combined with the vaccine. However, the final results and subsequent phases are awaited [63,65].

#### 5.1.3. Other Approaches: Heat Shock Proteins (HSP) & Personalized Neoantigenic Peptides

Several experimental vaccines are still under development. The approaches of these candidate therapies include heat shock protein (HSP) and personalized peptide vaccines. The HSP approach was designed to induce a highly specific anti-tumor inflammatory response by participating in antigen presentation of peptides to dendritic cells. A single-arm, non-randomized phase II trial in recurrent GB who received HSP peptide complex 96 (HSPPC-96) after surgery demonstrated a median OS of 42.6 weeks. Another study compared the effects of the HSPPC-96 vaccine + BEV versus BEV alone. However, the trial was suspended due to failure to get the primary point [21,57,66].

Personalized peptide vaccinations comprise up to 20 components, including personalized neoantigen peptides extracted from the patient’s resected tumor using whole-exome sequencing (WES) technology. This method aims to create reactive T cells that can infiltrate tumor cells and make them more susceptible to immunotherapies [52]. Recent phase I studies have yielded encouraging findings, demonstrating the viability of tailored vaccinations. According to recent phase I/Ib clinical trials, patients with GB can benefit from tailored peptide vaccinations with multi-epitope neoantigens. Patients who had already been surgically resected, radiated, and had not received dexamethasone were included. Results showed a specific CD4+ and CD8+ T-cell response, which developed into a memory genotype with enhanced tumor-infiltrating T cells. Ongoing pilot trials are already studying combinations of personalized neoantigen vaccines (NeoVax) with immune checkpoint inhibitors (CPIs), such as nivolumab or varlimumab [49,67,68,69].

### 5.2. Oncolytic Virus Therapy

**Figure 2:**
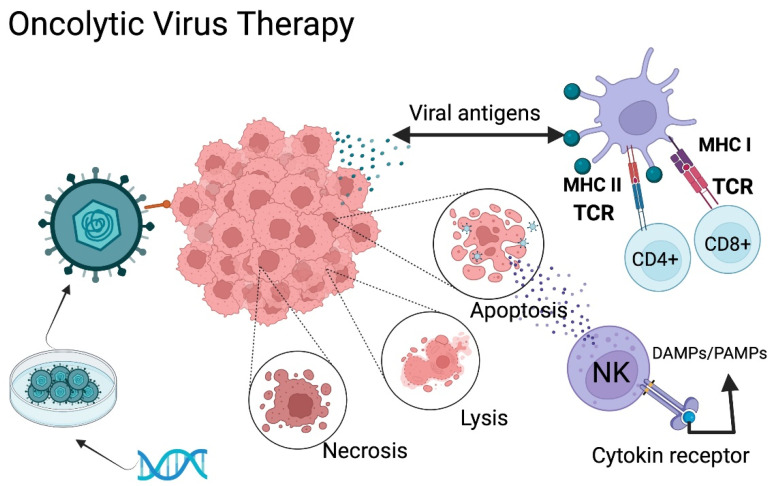
Oncolytic Virus Therapy Mechanism of Action. Oncolytic viruses (OVs) can be classified into two categories: natural viruses and genetically modified viruses. Modified viruses are loaded with specific receptors to recognize the GB through genetic engineering. The virus infects the tumor cells and generates either lysis, necrosis, or apoptosis, causing the release of tumor antigens, pathogen-associated molecular patterns (PAMPs), and damage-associated molecular patterns (DAMPs). The antigen-presenting cells present these antigens to T-lymphocytes, promoting activation of an adaptive immune response. TCR: T-cell receptor; NK: natural killer MHC I: Major histocompatibility complex class II. MHC II: Major histocompatibility complex class II. DAMPs: Damage-associated molecular patterns; PAMPs: Pathogen associated molecular patterns. Created with BioRender.com.

Employment of oncolytic viruses (OV) to mediate immune activity has strong potential in cancer treatment. OV can be classified into two categories: natural viruses and genetically modified viruses. However, the two categories share the same goal, selective infection of cancer cells and intra-tumoral action. At first, this therapy was developed for direct destruction of tumoral cells, but it has shown a wider range of potential applications. OV immunologic mechanisms include the release of tumor antigens (DAMPs), inhibition of tumoral immunosuppressive genes, transport of pro-inflammatory agents to tumoral cells, and tumoral microenvironmental disruption, among other factors that favor T-cell infiltration and a more effective immune response against the tumor (Figure 2) [18,49,63,70,71,72,73].

In normal cells, viral replication results in high protein kinase R (PKR) expression and activates the interferon signaling pathway, resulting in strong antiviral activity. However, most tumor cells have PKR deficiency, due to Ras pathway activation that reduces PKR, altering its function [72]. OVs selectively damage cancer cells through the inherent affinity of some viral receptors in the tumor cell surface, and through genetic engineering have added specific tumor receptors, such as EGFRvIII, PDGFR, IL-13R, or RGD peptide. These viruses are genetically modified to disable pathogenicity against healthy cells [70,73].

In addition, oncolytic viruses have a predilection for cancer cells, due to the inherent inability of these cells to have an adequate response to stress and homeostasis. The cellular signaling responsible for the detection of viral infection may be abnormal, making the viral “attack” more efficient [74]. Oncolytic viruses have not only demonstrated success, by directly combating cancer cells, but also directly influence the tumor microenvironment. As mentioned, the main mechanism of this therapy is the cancer cell death driven by the selective replication of oncolytic viruses, causing the release of tumor antigens, improving the inflammatory immune response [70,73].

#### 5.2.1. Herpes Simplex Virus

**Figure 3:**
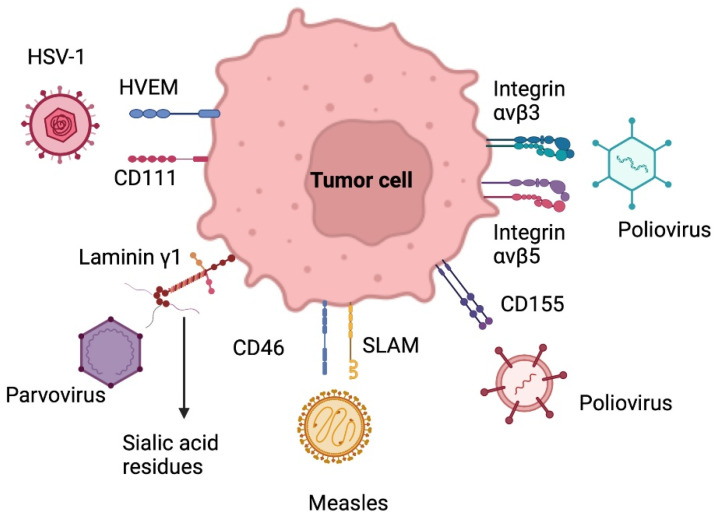
Target receptors of oncolytic virus therapy. HSV-1: Herpes simplex virus 1; HVEM: herpes virus entry mediator SLAM: signaling lymphocyte activation molecule. Created with BioRender.com.

Herpes simplex virus type 1 (HSV-1) is a double-stranded enveloped DNA virus, a member of the alpha herpes virus family. The entry of this virus into the central nervous system is due to the union of the viral glycoprotein D (gD) to the cell surface protein Nectin-1 (CD111) and through the herpes virus entry mediator (HVEM) (Figure 3). This virus has a direct lytic effect on tumor cells, as well as tumor destruction by tumor-specific immune responses. Neurotoxicity can be controlled through genetic attenuation. The oncolytic mutants that have been most studied to attack GB are dlsptk HSV, HSV-1716, G207, G47Δ, rQNestin34.5v., among others [74,75].

#### 5.2.2. Adenovirus

Adenoviruses are non-enveloped icosahedral viruses with a double-stranded DNA genome. The use of adenoviruses for gene delivery is known. The cellular tropism of human adenoviruses (HAd) differs between the different serotypes. Adenovirus serotype 5 (AD5) is a conditionally replicating adenovirus (CRad) and is the most widely used for gene delivery and the most studied [74,75].

In adenoviruses, DNX-2401, also known as delta-24 RGD, has shown effectiveness and safety for GB patients. This virus contains, by genomic modification, the RGD peptide (Arg-Gly-Asp) in its outer layer, which recognizes integrins (ανβ3 & ανβ5) present on the surface of many tumors, including gliomas, which allows cell entry through direct binding to tumor receptors. This modification has shown an enhanced anti-tumoral effect in cells with no RB pathway. Combined serotypes to increase the affinity of the virus to tumor cells and to attribute a survival mechanism to them are currently developing [56,74].

#### 5.2.3. Poliovirus

Poliovirus is a single-stranded, non-enveloped RNA virus and belongs to the picornaviridae family. The neurotoxicity of poliovirus and its usefulness for cancer therapy in GB lies in its selective binding to poliovirus receptors (PVR) in motor neurons (NECL-5 or CD155) (Figure 3). Furthermore, the upregulation of CD155/PVR has been demonstrated in GB samples, which may explain the particular tropism of an attenuated poliovirus strain (Sabin) for glioma cells [74].

Poliovirus is an extremely toxic virus for humans, so to reduce its virulence, it can be attenuated by replacing the viral internal ribosome entry (IRES) at the 5’ end of the viral RNA, with an IRES from the related human rhinovirus type 2 (HRV2). This modification is known as PVS-RIPO159, which has shown greater selectivity for GB cells. HRV2 IRES binds to the cellular double-stranded RNA-binding protein 76, and 45 kDa activated T-cell nuclear factor heterodimer (DRBP76–NF45), driving viral replication in glioma but not in healthy cells. In clinical trials, it has shown a safe and effective profile in malignant gliomas (NCT01491893) (NCT03043391) (NCT02986178) [70,74,75].

#### 5.2.4. Parvovirus

Parvovirus is a single-stranded DNA virus with an icosahedral capsule that belongs to the Parvoviridae family. The virus replicates in the nucleus and cytoplasm, binding to laminin γ1 on the glioma cell surface through silic acid residues. H1-PV (rat protoparvovirus H-1) binds to cell surface receptors and enters the cell by endocytosis. Viral DNA replication occurs when mitotic cells enter the S phase, damaging the DNA and destroying target cells. In addition, the clinical trials carried out have shown a safe and effective profile, with improvement in the recruitment of activated cytotoxic T lymphocytes in patients with malignant gliomas. It is a therapy that can be administered intravenously and intratumorally [70,75,76].

#### 5.2.5. Measles Virus

The measles virus is a paramyxovirus with a negative-stranded RNA genome. It uses the signaling lymphocyte activation molecule receptor to enter cells and replicates in the cytoplasm (Figure 3). Wild-type strains are very aggressive to humans, so oncolytic therapy is based on attenuated strains, including the Edmonston strain (MV-Edm) evaluated in clinical trials for GB, multiple myeloma, and ovarian cancer. Several mutations have been made in this strain, including the MV-CEA, which expresses the carcinoembryonic antigen. In order to trace the viral gene, this strain leads to apoptosis of glioma cells by the formation of syncytia. MV-NIS, another mutation of MV-Edm, expresses the human sodium iodide symporter (NIS) to track infection by isotopes. Other studies with measles strains have shown interesting results in mouse models of gliomas [74,75].

#### 5.2.6. Oncolytic Virus: Clinical Trials

Different vectors have been studied in OV therapy, including adenovirus (DNX-2401/Ad5-delta24-RGD), parvovirus (ParvOryx), measles (MV-CEA), HSV, polio (PVSRIPO), and replicating retroviral vectors (Toca 511), all of which have shown efficacy and safety in phase I and II clinical trials [18,49]. Only Toca 511 (vocimagene amiretrorepvec) therapy has been explored in a phase III clinical trial, with data showing a median OS of 14.4 months and an unusual complete response in 5 patients who lived for 33.9–52.2 months after receiving the therapy. A randomized, open-label, multicenter phase II/III trial (NCT02414165) testing Toca 511 treatment was also prematurely halted due to a lack of OS improvement [77].

Combined immunotherapy trials with oncolytic virus strategy are currently being conducted. An ongoing phase II combination trial (CAPTIVE [NCT02798406]) with intra-tumoral injection of DNX-2401 + anti-PD1 checkpoint inhibitor pembrolizumab in recurrent GB is the main example of the combination of two immunologic approaches. Preliminary results show adequate toleration and slight OS benefit, but final results must be awaited once the trial finishes in August 2023 [78].

Based on a non-randomized, open-label, dose-escalation phase I trial (NCT01491893) conducted in HGG, the FDA designated the intra-tumoral infusion of recombinant poliovirus (PVSRIPO) as a “breakthrough therapy” in May 2016. This innovative therapy had a median OS of 12.5 months, and 20% of patients are still living after 57-70 months after treatment. Following the first findings, a randomized phase II trial was established to assess PVSRIPO in patients with recurrent grade IV malignant glioma, which is now underway and expected to be completed in December 2023 [79]. Many recurrent GB phase I/II clinical trials are currently being conducted (Table 2). Current evidence had suggested OS improvement making oncolytic therapy promising. However, the clinical benefit should be tested compared to SOC in further randomized and controlled phase II/III studies for further conclusions.

### 5.3. Checkpoint Inhibitors (CPI´s) Therapy

Immune checkpoints are cell surface receptors that govern critical immune response activation pathways. In order to avoid autoimmunity, these receptors down-regulate immune cells and promote self-tolerance. On the other hand, as part of its immune evasion mechanism, cancer cells produce large levels of immunological checkpoints, such as the programmed death 1 receptor (PD-1) and its ligand (PD-L1).

Cancer promotes a chronic inflammatory state, which drives a hyperactive state of T cells. However, upon antigen persistence, the effector function of T cells starts to decline. Furthermore, this exhaustive state promotes the overexpression of inhibitory receptors and leads to an increase of regulatory T cells [51,80].

Key immune checkpoint inhibitors are involved during the regulation of effector T cell activity. These include cytotoxic T-lymphocyte antigen 4 (CTLA-4), CD28, mucin domain 3 (TIM-3), and indoleamine 2,3 dioxygenase-1, among others.

CD28 is mainly expressed on CD4+ T cells and regulates the activation of effector T cells through binding to the cofactor B7 [80,81]. CTLA-4, also named CD152, has a similar protein sequence as CD28; however, it has over 20 times more affinity for B7 than CD28, which leads to a competitive bind to B7 and inhibition of effector T cell activation. CTLA-4 expression is regulated by Foxp3 and nuclear factor of activated T cells (NFAT) on activated conventional T cells and regulatory T cells [80,81]. It is involved in the priming phase, because its ligands are predominantly expressed on APCs.

PD-L1 is a homolog 1 of B7 (B7-H1), which is expressed in tumor cells, APCs, B cells, and parenchymal cells [55,80]. This ligand increases its expression as a response to inflammatory cytokines, mainly interferons. By binding PD-L1 to its programmed death receptor 1, expressed on activated T cells, it induces apoptosis of T cells and promotes proliferative mechanisms of PD-L1 expressing cells [51,81,82]

**Figure 4:**
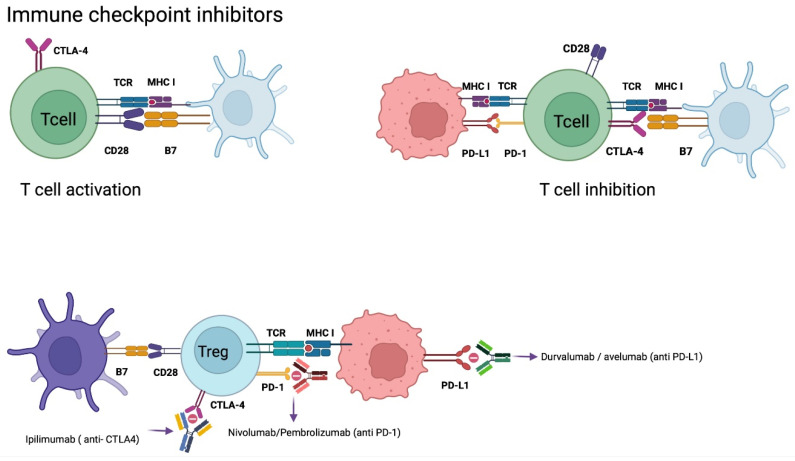
Checkpoint Inhibitors Mechanism of Action. Tumor cells evade the immune system as a defense mechanism. PD-L1 expression on tumor cells binds to PD-1 expressed on T cells, generating anergy of cytotoxic T cells. CTLA-4 expressed on T cells when it binds to B7 increases the expression of T-regs, generating an immunosuppressive response. CTLA-4 has a higher affinity for B7 than CD28 (B7 and CD28 when bound activate cytotoxic T cells). Monoclonal antibodies antagonize CTLA-4, PD-1, and PD-L1 preventing suppression of the immune response by cytotoxic T cells. Abbreviations: CTLA-4, cytotoxic T lymphocyte antigen 4; DC, dendritic cell; MHC, major histocompatibility complex; PD-L1, programmed cell death ligand-1; PD-1, programmed cell death-1; TCR, T-cell receptor; T-regs, regulatory T cells. Created with BioRender.com.

CPI treatment is a monoclonal antibody-based technique that targets immunological checkpoint receptors like PD-1 and PD-L1 to prevent immunosuppression and stimulate lymphocyte activation against tumoral cells [55,57,63,82]. This is a highly effective therapy for many cancers, such as renal carcinoma, non-small-cell pulmonary carcinoma, and melanoma. Recent studies observed the expression of PD-L1 in >80% of newly diagnosed GB and 72% of recurrent GB. Even though PD-L1 is present in GB, it is at a much lower level compared with renal cancer or melanoma [33]. Some of the current monoclonal antibodies that have been investigated are nivolumab, pembrolizumab, and cemiplimab (targeting PD-1), durvalumab, avelumab and atezolizumab (targeting PD-L1), and ipilimumab (targeting CTLA-4) (Figure 4) [83].

#### Nivolumab

Several phase I clinical trials in recurrent GB have been conducted to study anti-PD-1 checkpoint inhibitors, such as nivolumab alone or in combination with other CPI. Results show a slight increment of PFS and OS rates, as well as less toxicity when nivolumab is used alone. In a randomized, open-label phase III trial (CheckMate-143 [NCT02017717]), nivolumab was compared to BEV in recurrent GB, but the study was prematurely stopped due to lack of efficacy [78]. Nivolumab plus routine TMZ/RT is being tested in newly diagnosed GB patients with MGMT-methylated tumors (CheckMate-548 [NCT02667587]) and MGMT-unmethylated tumors in ongoing randomized, placebo-controlled phase III trials (CheckMate-498 [NCT02617589]). However, the data presented so far is dismal and predicts unsuccessful results (Table 3).

CPI therapy for GB seemed to be promising at first glance in the preclinical instance. However, clinical trials have not shown significant benefit in OS and PFS. Different target checkpoint inhibitors continue to be investigated, including CD47, CD24, CD37 (NCT02658981), LAG-3, and TIGIT/CD96 [83,84]. Additionally, combination with different immunotherapy strategies may be a future direction for CPI research.

### 5.4. Chimeric Antigen Receptor T Cell Therapy (CAR T)

**Figure 5:**
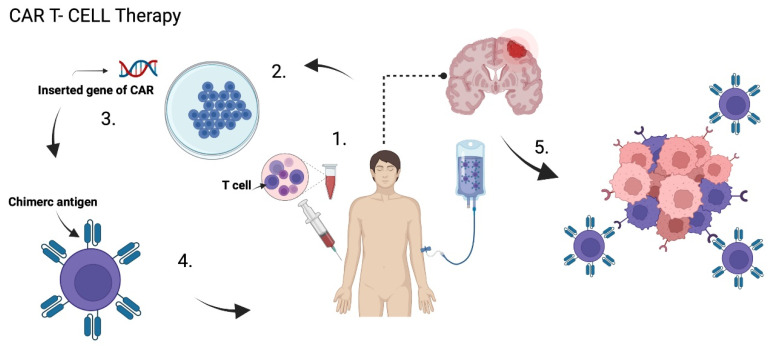
CAR T Cells Therapy. (1) T cells are extracted from the peripheral blood of patients, then (2) they are ex-vivo amplified and genetically remodeled so that (3) they express specific chimeric antigen receptor (CAR) in the cell membrane. (4) CAR T cells are injected back again in the patient, which can (5) specificallyrecognize the tumor cells and induce apoptosis. Created with BioRender.com.

Chimeric antigen receptors (CARs) are T lymphocytes genetically modified in a laboratory to express an artificial T lymphocyte receptor that targets specific tumor antigens. T cells are first obtained from the peripheral blood of patients, then, they are ex-vivo amplified and genetically remodeled so that they express specific receptors in the cell membrane. Then, CAR T cells are injected back again, either intravenously, intratumorally, or intracranially, so that they act on the tumor [61,82,85] (Figure 5). These CARs are composed of an extracellular antigen recognition fraction, mainly derived from a monoclonal antibody connected to a spacer domain, a transmembrane region, and an intracellular CD3ζ chain. Its intracellular domain allows the activation of T cells [86,87].

CAR T cells specifically identify cancer cells and lyse them, as long as the specific antigen is expressed on the cell membrane. They have the flexibility to recognize tumor antigens in carbohydrate, protein and glycolipid forms, without the presence of a major histocompatibility complex [61,86,87].

Histochemical analysis of GB tumors has allowed the identification of some treatment targets. However, the selectivity of CAR T cells and the heterogeneity of tumor antigen expression in GB complicates the performance of this therapy. With the appearance of second and third-generation CARs that include CD3ζ with 1 or 2 co-stimulatory domains, it is possible to combine several co-stimulatory proteins and domains to overcome GB heterogeneity. With the fourth generation, the antitumor potency is further improved by including additional proteins [82,87].

In GB, the CAR T cell target receptors studied are interleukin-13 receptor alpha 2 (IL13-Rα2) (NCT04003649), human epidermal growth factor receptor 2 (HER2), and epidermal growth factor receptor variant III (EGFRvIII) [88,89,90].

#### 5.4.1. HER 2

HER 2 is a transmembrane tyrosine kinase receptor involved in cell differentiation, proliferation, and adhesion. Its overexpression is intrinsically involved with the development of various tumors. It has been identified in approximately 80% of patients with GB [82,87]. In addition, HER2-CART cells have been shown to induce death in glioma cells “*en masse*”, including glioma-initiating cells (GIC), resulting in a significant antitumor activity in preclinical models. This approach has demonstrated a safe profile with no serious adverse events [89].

#### 5.4.2. EGFRvIII

As mentioned earlier in the text, EGFRvIII is a mutation of the extracellular domain of EGFR, which interferes with correct binding to the usual EGFR ligands. Therefore, it is associated with tumor progression and poor prognosis, specifically in GB. On the other hand, CAR T cells therapy against EGFRvIII has shown a safe profile, but no significant results in OS and PFS [88,90,91].

#### 5.4.3. IL-13Rα2

The IL13 monomer IL-13Rα2 is a cancer germline antigen that has activity in the PI3K/AKT/mTOR pathway, expressed in approximately 75% of GB and associated with a worse prognosis [44,47]. Brown et al. have studied the utility of IL-13Rα2-specific CAR T cells, observing a safe profile without significant positive results. A study evaluating the safety of IL13-Rα2 in combination with CPI therapy in GB is currently underway, with results expected by the end of 2022 (NCT04003649).

Applying this novel therapy to GB is still under intense ongoing research. Many clinical trials (Table 4) are testing the described promising targets, such as EGFRvIII (NCT02209376), EphA2, HER2 (NCT01109095), IL-13Ra2, and PD-L1, which are currently being executed. Recently, three of these antigens have been recognized in almost 100% of GB, which is an encouraging result. Even though preliminary results, and data obtained from previous studies, have not demonstrated some clinical benefit yet, evidence of CAR T cells infiltration on tumoral tissue has been observed, which proves the capacity of crossing the BBB of this novel therapy [21,49,87].

As previously stated, GB has multiple immunological escape mechanisms as well as a great immunoediting adaptive capacity, which poses a considerable challenge for CAR T cell therapy development. Nonetheless, this approach’s promising first outcomes open the possibility of designing and developing clinical studies combining CAR T cell therapy with any other immunotherapy technique and delivery method [55,87].

#### 5.4.4. CAR NK Cell Therapy

Natural killers (NKs) are lymphoid cells involved in the innate immune system and can be identified by CD3(-) CD56(+). They receive activation and inhibitory signals through germline-encoded receptors. NKs do not require prior antigen presentation, as they can recognize cells whose MHC I molecular expression is compromised. When the damaged cells are identified, NKs act by antibody-dependent cell-mediated cytotoxicity (ADCC) through FcγRIIIA/CD16a. The attack mechanisms by NKs are through the release of perforins and granzymes, inducing apoptosis through the caspases pathway by regulating death ligands, such as FAS and TRAIL [92,93].

NKs infiltrate approximately 90% of GBMs. However, these cells are suppressed due to the immunosuppressive environment created by the tumor. For these reasons, CAR NK therapy has been studied to potentiate the immune response of NKs on the tumor. The similar domains used in CAR-T cells have demonstrated adequate functionality in NKs. Additionally, the combination of CD244 (SB4), DAP12, or DAP10-derived signaling domains demonstrated potentiated activity [94,95]. Furthermore, advantages of CAR NK cells over CAR T cells have been proposed, including a lower risk of side effects [93,95].

Preclinical studies with CAR-NK cells have used first and second-generation CAR designs. CAR NK-92 is based on NK-92 cells, which specifically target the receptors EGFR and EGFRvIII. In mouse models, CAR NK-92 and CAR NK showed increased cytotoxicity, IFN-γ secretion, exhibited benefit in xenograft survival, and better survival response with CAR-NK-92 [94,95]. So far, the only human cell line approved for treatment in humans is NK-92. A phase I clinical trial (NCT03383978) with 30 patients is currently underway in Germany to measure the maximum tolerated and maximum safe dose of CAR NK-92 in patients with HER2-positive recurrent glioblastoma. The first results are expected by the end of 2022 [93]. Currently, the scope for the development of CAR NK cell therapy for gliomas is wide. Although NK cell has the physiological advantage of having its own specific mechanism against tumors, CAR NK cell therapy has lagged behind CAR T cell therapy since the latter has been further explored. The results in early preclinical studies have been encouraging. In the coming years, the first results of clinical trials will lead to further research on new generations of CARs in NKs.

## 6. Future Perspectives: Synthetic Molecules and Natural Compounds

### 6.1. Synthetic Molecules

In the last decade, a vast amount of knowledge has been gathered regarding the molecular characteristics of GB, e.g., alterations in signaling pathways, cellular microenvironment, immunogenicity, among others. Likewise, a better understanding of the mechanisms of cell damage and cell death induced by radiotherapy and chemotherapy has been obtained, which has allowed the development of novel therapeutic approaches involving these mechanisms to cause tumor reduction and potentiate existing therapies. In addition, several synthetic molecules have been developed and tested in the preclinical stage. Here we detail some of the most promising therapies for GB.

#### 6.1.1. RES-529

The PI3K/AKT/mTOR signaling pathway stimulates a variety of cellular processes such as transcription, translation, and cell survival. The common path for PI3K/AKT pathways is the activation of mTOR complexes constituted by mTORC1 and mTORC2, which are serine-threonine kinases that modulate several cellular processes involved in survival, proliferation, apoptosis, and autophagy [96]. A precise description of the PI3K/AKT/mTOR pathway is not the objective of the present article and can be revised elsewhere.

Regarding mTORC1, it is modulated by several pathways, such as AKT, RAS-ERK, and AMPK, to name a few. The latter regulates mTORC1 under stress conditions, such as hypoxia or DNA damage. Unlike mTORC1, the activation of mTORC2 is not as well described. However, this complex plays an essential role in regulating cell survival. This pathway is relevant during angiogenesis by inducing the expression of VEGF ligand and receptor via hypoxia-inducible factor 1α (HIF-1α) mechanisms. Genetic mutations in PI3K/AKT/mTOR are present in up to 80% of GBs, leading to the pathway’s constant activation and may explain the invariable recurrence after treatment [96].

The only currently approved mTOR inhibitors are rapamycin analogs, used for renal cell carcinoma (everolimus) and pancreatic tumors (temsirolimus). These agents only inhibit mTORC1, but not mTORC2, which is the reason why they have shown limited clinical efficacy in other tumor types, such as GB. Recently, the small molecule RES-529 (Palomid 529) has emerged as the first PI3K/AKT/mTOR pathway inhibitor targeting both mTORC1 and mTORC2 through mTOR complex dissociation. RES-529 is an oral compound with a lack of affinity to drug efflux transporters ATP-binding cassette subfamily B member 1 and subfamily G member 2 (ABCB1 & ABCG2), which provides an effective BBB penetration. The first insights into RES-529 mechanisms emerged from in vitro experimental studies on neuroblastoma cells [96]. Safety and tolerance have been evaluated in two phase I open-label trials in patients with neovascular age-related macular degeneration (NCT01271270 and NCT 01033721), which was well tolerated with no drug-related systemic adverse events reported [96,97].

In vitro experimental studies showed that RES-529 increased GSK-3β activity promoting apoptosis by reducing *survivin* expression. Furthermore, a synergic effect with RT was described to further increase GSK-3β activity and decrease *survivin* levels. Another in vitro observed effect of RES-529 is a dramatic reduction in cell cycle-associated proteins, such as cyclin B1, cyclin D1, cyclin-dependent kinase 4 (CDK4), and CDK6. Cellular and mouse models showed dose-dependent antiangiogenic effects by inhibition of VEGF and β fibroblast growth factor (β-FGF) through the reduced activity of the PI3K/AKT/mTOR pathway. Significant growth inhibition in cancer cell line studies was observed, as well as in animal models, where an approximate 70% decrease in tumor volume was observed with dose-dependent behavior [96,97].

Synergistic activity of RES-529 with RT was first observed in prostate cell and animal models. The combination therapy showed an increase in autophagy, apoptosis, tumor senescence, DNA damage, reduction in tumor volumes, and delay in median time to progression. Gravina et al. studied the combination of RES-529 + BEV or sunitinib in GB cell lines, in vivo subcutaneous xenografts, and orthotopic intra-brain cell inoculation. In agreement with other studies, they observed an induced G2/M cell cycle accumulation and apoptosis. They also found an increased efficacy of RES-529 when combined with the antiangiogenic agents BEV or sunitinib, with a significant reduction of tumor growth and volumes in animal models [96,97].

The oral formulation of RES-529 has received orphan-drug designation by the US Food and Drug Administration, due to the promising results of RES-529 on experimental models. This novel therapy is currently being evaluated in various clinical trials for GB. A major concern is the potential for increased adverse events in combination therapies because of dual inhibition of the mTOR complex. Therefore, clinical trials evaluating RES-529 in GB are further needed to elucidate safety, efficacy, and the potential synergy with antiangiogenic agents, such as BEV, mainly in the recurrence scenario [96,97].

#### 6.1.2. ATX-101

During the DNA replication process, the machinery of numerous proteins is required, which constantly interact with the proliferating cell nuclear antigen (PCNA). PCNA is a scaffold protein belonging to the conserved DNA sliding clamp family of proteins. It is essential for DNA’s vital processes like replication, repair, epigenetic modification, damage tolerance, chromatin remodeling, and cell cycle control. The specific proteins required for each DNA process interact with PCNA through either of two PCNA-interacting motifs: (1) The PCNA-interacting peptide (PIP) sequence, called the PIP box, or (2) The AlkB homologue-2- PCNA-interacting motif (APIM). Most of the proteins required in DNA essential processes interact with PCNA through the high-affinity canonical PIP box in physiological conditions, contrary to proteins involved in stress management, which have lower affinities for the canonical PIP box and higher affinity for APIM or non-canonical PIP boxes, due to the post-transductional modifications of PCNA induced by DNA damage. Multiple proteins involved in cell signaling through MAPK and PI3K/Akt/mTOR pathways also contain a putative PCNA-interacting motif, mainly APIM [98,99].

ATX-101 is a cell-penetrating peptide containing APIM, which selectively targets modified PCNA in stressed cells, affecting its cytoplasmic and nuclear roles in cell signaling and DNA repair. It has been demonstrated to disrupt the ability of tumoral cells to repair and tolerate DNA damage caused by chemo or radiotherapy, which has proven to induce apoptosis, reduce Akt/mTOR signaling, and stem cell phenotypes of GICs in both in vitro and in vivo experiments [98,99].

As previously mentioned, RT represents a pillar of GB management by inducing various cell death types, such as apoptosis, necrosis, necroptosis, and autophagy. These are the result of cytotoxic effects mediated by the induction of single- or double-stranded DNA breaks (SSBs or DSBs) that exceed the damage tolerance of DNA repair. After a DSB is produced, histone H2AX is rapidly phosphorylated (γH2AX), marking the damaged area that needs to be repaired. Radiosensitivity or radio-resistance in a cell is associated with the response capacity to DNA damage. In GB, the adaptative radio-resistance of the tumor cells almost invariably leads to recurrence. The experimental peptide ATX-101 was proven to enhance the effect of CT/RT in a clinical phase I study in advanced bladder tumors. An experimental study of in vitro GB cell models showed a dose-dependent radio-sensitizing effect of ATX-101, and increased levels of (γH2AX) after combined RT + ATX-101 therapy, which suggests increased levels of DSBs and reduced ability to DNA-repair. This behavior was observed in much lower levels in models treated with ATX-101 only [99].

GB’s heterogeneous cell population includes glioma-initiating cells (GICs), which show a glioma stem-cell (GSC) phenotype characterized by Sox2, CD44, CD90, and OCT3/4. These cells are located in tumor areas with elevated hypoxia and can infiltrate surrounding tissue, which constitutes the leading cause of recurrences. The stemness of GSC is modulated to a great extent by the Akt/mTORC1 pathway, which is a primary target of ATX-101. In vitro experiments have proved a dose-dependent reduction of the expression of mesenchymal and stem cell markers, such as CD44 and CD90, with ATX-101 treatment, and increased glial differentiation, suggesting a partial reversion of the pro-neural to mesenchymal transition characteristics of GICs [99].

Gravina et al. observed a reduction in tumor growth in several animal models, including intracranial tumor models. They attributed the results to the inhibition of the nuclear and cytoplasmic roles of PCNA by ATX-101, causing altered DNA-repair mechanisms, radiosensitivity, reduced stemness, and apoptosis [99].

The evidence suggests that the drug ATX-101 is a novel therapeutic approach for GB. Further phase I/II clinical studies are encouraged to evaluate the safety and efficacy of combination therapy with RT + ATX-101. Infiltration of tumoral cells in healthy tissue remains a concern for recurrence, and novel ATX-101 mechanisms do not elucidate an effect on this important aspect.

#### 6.1.3. GLPG1790

As previously commented, GB initiates from a small population of GSCs named GICs that will proliferate. Once a tumor is formed, tumor necrosis is increased, despite abundant angiogenesis, which promotes highly hypoxic tumor areas. The hostile environment recruits endothelial cell precursors, and more glioma stem cells (GSCs) that rapidly grow and infiltrate healthy tissue, determining the invariable tumor recurrence and chemo-radio resistance. Standard of treatment in GB induces cell death, mainly on differentiated cancer cells. For that reason, a forced differentiation of GICs by activating endogenous pathways could increase the efficacy of conventional therapies and reduce resistance and tumor recurrences [100].

Erythropoietin-producing hepatocellular (EPH) proteins are a large family of receptors with tyrosine kinase activity that promote malignancy and stemness in GB. These receptors include Eph-A (A2 & A3) and Eph-B (B4), the binding ligands of which are Ephrin-A and Ephrin-B, respectively. Its activation triggers a complex network of signaling that plays a crucial role in GB progression and glial/neuronal differentiation. EphA3 is commonly overexpressed in the most aggressive mesenchymal subtype and GICs, maintaining an undifferentiated phenotype by modulating MAPK signaling. Infiltrative invasion of GICs is promoted by co-expression of EphA2 and EphA3. These receptors regulate several processes related to GB progression, such as angiogenesis and local invasion [100].

GLPG1790 is the first small molecule of oral administration with inhibition activity against diverse Eph receptor kinases, demonstrated to force differentiation of GSCs in GB preclinical models. Gravina et al. tested the effectiveness of GLPG1790 in preclinical in vitro and in vivo GB models. They observed down-regulation of mesenchymal marker expression (CD44, Sox2, nestin, OCT3/4, Nanog, CD90, CD105) and up-regulation of GFAP, βIII tubulin, and pro-neural markers. These were mediated by a complete blockade of EphA2 receptor signaling due to GLPG1790 oral administration. That resulted in anti-glioma effects, such as reduced tumor growth in vivo models [100].

Forced differentiation of GSCs is a promising novel approach in cancer treatment, which can be applied to GB. GLPG1790 induces differentiation of GSCs and produces antitumoral effects, even superior to RT alone. The mentioned results in preclinical phases support the investigation of GLPG1790 as a promising therapy for phase I clinical trials on GB. However, further preclinical studies are needed to elucidate possible interactions between RT/CT and GLPG1790 [100].

### 6.2. Natural Compounds

#### 6.2.1. Trans-Sodium Crocetinate

Crocetin is a main active ingredient of a spice named saffron, derived from the flower of Crocus sativus and fruits of *Gardenia jasminoides*. Saffron is considered the most expensive spice in the world. To fully optimize the medical utility of crocetin, trans-sodium crocetinate (TSC) has been developed, which is a sodium salt of crocetin. Its mechanism of action is to improve oxygen diffusion from plasma to tissue through alteration of water structure [101].

The plasma thickness and solute concentration gradients are two crucial factors in oxygen diffusion physics. Crocetin owns a rigid hydrophobic structure in its interior, repelling water molecules and a pair of polar heads at each end of the molecule, facilitating an ordered structure of water molecules in plasma via H-bonding. This process directly impacts on the diffusivity of solutes by a notable reduction in the plasma fluid density [101].

Solid tumors usually exhibit hypoxic components, which is a main cause of radio-resistance. Due to the TSC anti-hypoxia mechanism, it has been tested as a potential treatment for tumors such as GB. The improvement in oxygen diffusion has demonstrated anti-tumoral effects in preclinical studies through antiproliferative, pro-differentiation, anti-cell cycle progression, growth, and immune modulation mechanisms. This natural compound has been observed to improve radiotherapy results in GB trials, probably due to an increased oxygen diffusion that promotes radio sensibility in previously hypoxic cells [101,102].

In the clinical stage, Gainer et al. conducted a multi-center, open continued phase I-II trial, single-arm design, that included 59 patients, to determine the safety and efficacy of adding TSC to RT during standard of care therapy. During the trial, 27 tumor-bearing patients regressed, and combined TSC + SOC treatment resulted in a tumor volume reduction of >40%. The most impressive result in this trial was the complete disappearance (100% reduction) of the tumor in 30% of the patients (11 patients of 59). The outcomes of this trial strongly support the safety and beneficial effects of TSC in combined therapy with RT for GB treatment [103].

Safety and tolerability are the major considerations when proposing a new drug treatment. TSC + RT therapy safety was evaluated in two clinical trials (INTACT NCT00339300 & NCT 01465347) [103]. The INTACT study [NCT 03393000] was a phase III clinical trial that evaluated the safety and efficacy of TSC in newly diagnosed GB, but it was finished earlier because of a business decision on behalf of the sponsor. In the clinical trial conducted by Gainer et al. no serious adverse events were associated with TSC in any patient [101,102,103]. Therefore, it seems that adding TSC to SOC increases the toleration of patients to TMZ treatment at any age. To date, TSC has proven to be a very safe and well tolerated drug in three clinical trials, probably due to its purely physical–chemical mechanism of action [101,103]. Thus, efficacy studies comparing TSC + SOC versus SOC alone must be conducted to elucidate the radio-sensitizer potential of TSC, which could represent a radical change in GB treatment.

#### 6.2.2. PBI-05204 (Oleandrin)

Oleandrin is a cytotoxic cardiac glycoside that constitutes the major component of the botanical drug PBI-05204, which is a modified supercritical carbon dioxide extract of Nerium oleander plant leaves. This drug has undergone both phase I and phase II clinical trials as a potential treatment for a variety of cancers, such as melanoma, lung, osteosarcoma, prostate, breast, and pancreatic cancer, showing safety in its oral administration, as well as specific activity versus malignant cells [104,105].

The proposed antitumoral activity of PBI-05204 is by down-regulation of the PI3k/AKT/mTOR pathway, the caspase 9 pathway activation, the inhibition of NF-kβ activation, and a potential radio-sensitizing effect. Previous pre-clinical studies using in vitro and in vivo models have demonstrated dose-dependent effectiveness in reducing tumor progression, increasing FPS, OS, and radio-sensitivity. The addition of PBI-05204 to conventional RT significantly improved the anti-proliferative and antitumor activity of RT in GB cells of in vitro and in vivo models [104,105].

PBI-05204 potential radio-sensitizing mechanisms are suggested to inhibit DNA repair after damage induced by radiation, which promotes apoptosis, reduction in autophagy, and stemness inhibition [104,105]. Besides the proposed mechanisms, further research on PBI-05204 radio-sensitizing effects should be conducted to understand its potential effect in combination with RT for GB treatment. Exhaustive evaluation of PBI-05204 could lead to a natural, low cost and effective adjuvant therapy for GB treatment.

## 7. Discussion

The state-of-the-art in glioblastoma treatment is the result of many years and much effort in clinical research. The actual treatment lines are based on three pillars: surgical resection, radiotherapy and chemotherapy. Only a few important considerations have arisen since Stupp et al. introduced the standard of care more than fifteen years ago. These considerations include prioritizing quality of life and neurological function over maximizing resection during surgery. This can be achieved with novel tools, such as intraoperative function monitoring, neuro-navigation, and fluorophore drugs, allowing the patient to overcome the next phases of treatment. In addition, it has been confirmed that chemoradiotherapy outcomes depend on IDH mutation and MGMT methylation status, which are related to the repair response to DNA damage caused by ionizing radiation and alkylating agents.

Despite rigorous studies, temozolomide remains the main election for chemotherapy in newly diagnosed GB. However, lomustine mechanisms on MGMT enzymatic activity have been exhaustively studied, and it seems to be a promising combination therapy with temozolomide, enhancing its efficacy.

RT has a crucial role in newly diagnosed GB management since SOC was first described. Its main limitation lies in several radio-resistance mechanisms developed by tumor cells and the microenvironment, principally after the first RT course. This response reduces the benefits of re-irradiation after tumor progression. Recently, new synthetic molecules, such as ATX-101, and natural compounds, such as trans-sodium crocetinate (TSC) and PBI-05204, have demonstrated radio-sensitizer properties in pre-clinical and clinical trials, which represent a promising approach to enhance RT efficacy.

ATX-101 is a novel synthetic therapeutic molecule for GB with important mechanisms of stem cell modulation and disruption of DNA repairing processes, which have potential radio-sensitizing implications. Further phase I/II clinical studies evaluating the safety and efficacy of combination therapy with RT are encouraged. About natural compounds, PBI-05024 has proven to inhibit DNA repair after radiation-induced damage, induce apoptosis, and inhibit stemness, which are suggested radio-sensitizing mechanisms that should be explored in combination with RT at clinical stages. Nevertheless, the most promising natural molecule at the pre-clinical stage is TSC, the purely physical mechanisms of which have the potential to improve radiotherapy effectiveness through increased oxygen diffusion to hypoxic cells. This drug has proven to be safe and well-tolerated in combination with RT in phase I and II clinical trials, where the combination also induced complete tumor regression in some patients. An exhaustive evaluation of these alternatives could lead to a natural, low cost and effective adjuvant therapy for GB treatment.

In the last decade, bevacizumab and TTFs have been added to the treatment scheme for GB. BEV systemic therapy improves PFS without improving surveillance and has demonstrated an important reduction in radio-necrosis incidence. Although the FDA has approved BEV in some regions of the world, its effects on symptoms and quality of life are still a matter for debate. More randomized studies are required to clarify its role in highly symptomatic patients. About TTFs, several clinical trials have demonstrated the benefits of this therapy in newly diagnosed and recurrent GB, which is now considered a feasible alternative. Despite the evidence, disagreements over the conduct and design of the studies remains open to debate concerning the limitations of the technique. The costs and poor accessibility of TTFs represent additional challenges for its regular application in most neuro-oncologic centers.

Immunotherapy has been exhaustively tested for many years. The heterogeneity and immunosuppressive microenvironment of GB are the main challenges for immunotherapy, which may be overcome by employing synergistic mechanisms between therapeutic approaches that potentiate immunologic response against GB. A combination of therapeutic approaches, mechanisms of action, and delivery methods may be the next step in clinical research for GB treatment. Examples of combinations already tested include CAR T cell therapy plus checkpoint inhibition or temozolomide, TSC plus RT, DVRIPO, or DNX-204 oncolytic virus delivered via direct injection into the brain parenchyma, to mention a few. In addition, personalized immunologic mechanisms could be a potential option for CART-T cell therapy.

Stem cells have a crucial role in GB pathogenesis, malignancy, and resistance. The demonstrated effects in preclinical studies of new synthetic molecules and natural compounds have opened a research line on GSC inhibition therapies for induced differentiation strategies, which are future perspectives that may enhance RT and CT effects on GB.

## 8. Conclusions

Glioblastoma is a biologically heterogeneous and highly complex neoplasia that represents a major challenge for neuro-oncology research. Despite great efforts in therapy research, the actual management remains based on maximal safe resection, radiotherapy, and chemotherapy. The multiple resistance mechanisms, the immunosuppressive microenvironment, and tumor infiltration represent the main limitations to the standard of care. Combined therapeutic strategies and delivery methods, including immunotherapy, synthetic molecules, natural compounds, and glioma stem cell inhibition, may potentiate the standard of care therapy and represent the next steps in GB management research.

## Figures and Tables

**Table 1:** Vaccine Therapy.

Clinical Trial	Vaccine Studied	Description	Features	Primary Outcome & Overall Objective	Significant Result
ACT IV NCT 01480479	Vaccine against EGFRvIII Rindopepimut (CD-110)	Rindopepimut + TMZ in newly diagnosed EGFRvIII positive patients	Phase III 745 participants Randomized Parallel Assignment Double-blind Controlled	Compare OS in patients when treated with Rindopepimut + TMZ vs TMZ and control.	No significant difference in OS in minimal residual disease (MRD) (20.1 (95% CI 18.5–22.1) CI 18.1–21.9 vs. 20 months) and in significant residual disease (SRD) (14.8 [95% CI 12.8–17.1] vs 14.1 months [12.6–15.7] No significant difference in PFS in MRD (8.0 95% CI 7.1–8.5 vs. 7.4 months CI 6.0–8.7) HR 1.01 *p* = 0·91 And in SRD (3.7 months, 3.5–5.8 vs. 3.7 months, 3.3–4.9; 0.86, 0.66–1.12; *p* = 0.28)
NCT00045968 DCVax^®^-L	Dendritic cells vaccine DCVax^®^-L	DCvax-L in newly diagnosed GB following resection	Phase III 348 participants Randomized Parallel Assignment Double-blind Controlled	Compare PFS between patients treated with DCVax-L and control patients.	PFS has not yet been evaluated for this publication (will be analyzed later). Only OS result of the combined arms reported until now.
ICT -107 NCT01280552	Dendritic cells vaccine ICT-107	ICT-107 + maintenance TMZ in newly diagnosed GB	Phase II 124 participants Randomized Double-blind Controlled	OS Compare OS in patients when treated with ICT 107 versus Placebo DC.	ICT-107 was well tolerated. No significant difference in OS (17.0 (CI: 13.68–20.61) vs. 15.0 months (CI: 12.33–23.05) (HR = 0.87; *p* = 0.58) PFS was significantly better in patients treated with ICT-107 (11.4 vs. 10.1 months (HR = 0.64; *p* = 0.033).
NCT01814813	Heat shock protein (HSP) vaccine HSPPC-96	-	Phase II 90 participants Randomized Parallel Assignment Open label	Compare OS between HSPPC-96 + BEV vs BEV alone.	OS for the HSPPC-96 treated groups was 7.5 vs. 10.7 months for bevacizumab alone (HR = 2.06 [95% CI 1.18–3.60], p = 0.008).
NCT03018288	Heat shock protein (HSP) vaccine HSPPC-96	RT + TMZ and pembrolizumab +/− HSPPC-96 vaccine in newly diagnosed GB	Phase II 90 participants Randomized Parallel Assignment Double Blind	Determine whether the 1-year OS is improved in newly diagnosed MGMT unmethylated GB patients treated with RT + TMZ + Pembrolizumab followed by Pembrolizumab + TMZ +/− HSPPC-96 x 6 cycles	Ongoing study, estimated study completion date: 9 January 2025
NCT02287428	Personalized neoantigen vaccine NeoVax	NeoVax) + RT + Pembrolizumab in newly diagnosed GB	Phase II 56 participants Randomized Parallel Assignment Open Label	Adverse effects Number of participants clinically able to initiate post RT-vaccine therapy Number of participants with at least 10 actionable peptides.	Estimated Primary Completion Date: January 2025 Estimated Study Completion Date: January 2026
NCT02287428	Personalized neoantigen vaccine NeoVax	NeoVax + RT in newly diagnosed GB	Phase I/Ib 8 participants Non-randomized Parallel Assignment Open Label	Safety and tolerability.	Personalized vaccination therapy with multi-epitope neoantigens is feasible for patients with glioblastoma and increase immune response and the number of tumor infiltrating T cells.
NCT04015700	Personalized neoantigen vaccine GNOS-PV01 + INO-9012	GNOS-PV01 + INO-9012 in newly unmethylated GB	Phase I 12 participants Non-randomized Single Group Assignment Open Label	Dose-limiting toxicity. identify candidate tumor-specific neoantigens	Estimated Study Completion Date: 31 July 2023
NCT02149225 (GAPVAC)	Personalized neoantigen vaccine APVAC1 APVAC2	APVAC1 and APVAC2, GM-CSF and Poly-ICLC and TMZ in newly diagnosed GB	Phase I 16 participants Non-randomized Single Group Assignment Open Label	Patient-tailored safety of APVAC when administered with TMZ. Number of adverse events Frequency of CD8 T cells specific for APVAC peptides	Increased immune response and increased infiltration of T cells into the tumor with a balanced immune response. OS 29 months PFS 14.2 months
NCT03223103 (ATIM-31)	Personalized neoantigen vaccine Mutation-derived tumor vaccine (MTA)	MTA+PolyICLC+TTTFields in GBM	Phase I 13 participants Non-randomized Single Group Assignment Open Label	Dose-limiting toxicities	The vaccine is well tolerated and there were no unexpected adverse effects. Estimated Study Completion Date: May 2023
NCT02924038	Monoclonal Antibody CDX-1127 (Varlilumab)	Varlimumab (CDX-1127) + IMA950/polyICLC in newly diagnosed GBM	Phase I 14 participants Randomized Parallel Assignment Open Label	Adverse events Immune response of CD8 and CD4 in pre and post vaccine	Estimated Study completion Date: 31 December 2022

This table summarizes the most important research projects in vaccine therapy.

**Table 2:** Oncolytic Virus Therapy.

Clinical Trial	Oncolytic Virus	Description	Features	Primary Outcomes	Significant Results
NCT0241416 TOCA FC (flucytosine)	TOCA 511 retroviral replicating vector encoding cytosine deaminase	Toca 511 + Toca FC vs. lomustine, TMZ, or bevacizumab in recurrent HGG	Phase II/III 403 participants Randomized Parallel Assignment Open Label	Compare OS OF TOCA 511 + TOCA FC vs. standard of care after tumor resection for recurrence of HGG.	The study was stopped because did not improve OS (11.10 months vs. 12.22 months HR, 1.06; 95% CI 0.83, 1.35; *p* = 0.62).) or other efficacy endpoints.
NCT01470794	TOCA 511 TOCA FC	Toca 511 + Toca FC in recurrent HGG	Phase I 58 participants Non- randomized	Dose Limiting Toxicities Single Group Open Label	Toca 511 and Toca FC is tolerable and safe.
NCT02197169 (TARGET I)	DNX-2401	DNX-2401 ± interferon gamma (IFN-γ) for recurrent glioblastoma	Phase I 37 participants Randomized Parallel Assignment Open Label	Objective response rate (ORR) determined by MRI scan review.	DNX-2401 was well tolerated as monotherapy Poor tolerability of IFN.
NCT00805376	DNX-2401D	DNX-2401 (conditionally replication-competent adenovirus) +/− surgery in recurrent HGG	Phase I 37 participants Non-randomized Single Group Assignment Open Label	Maximum Tolerated Dose (MTD) of DNX-2401	DNX-2401 replicates and spreads within the tumor, generating direct virus induced oncolysis in patients. Median OS was 9.5 months regardless of dose. Five patients survived >3 years in the single DNX-2401 intratumoral injection group.
NCT02798406 (CAPTIVE)	DNX-2401	DNX-2401 + pembrolizumab in recurrent GB	Phase II 49 participants Non-Randomized Single Group Assignment Open label	Objective response rate (ORR)	DNX-2401 followed by pembrolizumab is well tolerated.Expected completion date August 2023
NCT03896568	Ad5-DNX-2401	Asses best dose and side effects of DNX-2401 in treating patients with recurrent HGG	Phase I 36 participants Non-randomized Sequential Assignment Open Label	MTD and adverse events	Estimated Study Completion Date: 31 May 2022
NCT01956734	DNX-2401	DNX-2401 + temozolomide in recurrenct GB	Phase I 31 participants Single Group Assignment Open Label	Adverse events. Tolerance of the combination of DNX-2401 and temozolomide	Completed, no results available
NCT03714334	DNX-2440	DNX-2401 in first or second recurrence of GB	Phase I 24 participants Single Group Assignment	Treatment related adverse events	Estimated primary completion Date: April 2022 Estimated Completion Date: October 2022
NCT02986178	PVSRIPO (oncolytic polio/rhinovirus recombinant)	PVSRIPO in recurrent grade IV glioma	Phase II 122 participants Single Group Assignment Open Label	Objective Radiographic Response Rate at 24 and 36 months.	Estimated Primary Completion Date: August 2023 Estimated Study Completion Date: December 2023
NCT01491893	PVSRIPO (oncolytic polio/rhinovirus recombinant)	PVSRIPO in HGG	Phase I 61 participants Non Randomized Sequential Assignment Open Label	MTD of PVSRIPO Number of participants Who Experienced Dose-Limiting Toxicities	OS was higher at 24 and 36 months
NCT00390299	Carcinoembryonic Antigen-Expressing Measles Virus (MV-CEA)	MV-CEA in treating patients with GBM	Phase I 23 participants Non Randomized Parallel Assignment Open Label	Dose-Limiting Toxicity Events MTD Grade 3+ adverse events	No dose limiting toxicities
NCT03294486	TG6002	Safety and efficacy of the oncolytic virus armed for local chemotherapy, TG6002/5-FC, in recurrent GBM	Phase I 78 participants + 24 participants in Phase IIa Sequential Assignment Open Label	Dose Limiting Toxicities Number of patients without documented tumor progression at 6 months	No results available
NCT03152318	oncolytic HSV-1 (rQNestin)	rQNestin34.5v0.2 + cyclophosphamide in recuurent HGG	Phase I 56 participants Non randomized Sequential Assignment Open Label	MTD of rQNestin34.5v.2 injected into recurrent malignant gliomas, with or without previous immunomodulation with cyclophosphamide.	Ongoing study Estimated Study Completion Date: December 2023
NCT01301430	H-1 parvovirus (ParvOryx)	Safety, tolerability and efficacy	Phase I/IIa 18 participants Single Group	Safety and tolerability Assignment Open Label	ParvOryx was safe and well tolerated. PFS was 111 days Median OS was 464 days.
NCT02062827	Second generation oncolytic herpes simplex virus (M032) (NSC 733972)	Safety, tolerability of the maximum dose of M032 in patients who would not be eligible for surgical resection of recurrent glioma.	Phase I 24 participants Single Group Assignment Open Label	MTD	Estimated Primary completion date: September 2022 Estimated Study completion Date: September 2023

This table summarizes the most important research projects in Oncolytic Virus Immunotherapy.

**Table 3:** Checkpoint Inhibitors Therapy.

Clinical Trial	Checkpoint Inhibitor Studied	Description	Features	Primary Outcomes	Significant Results
NCT02017717 (Checkmate 143)	Immunoglobulin 64 monoclonal antibody targeting the programmed death -1 (Pd-1) immune checkpoint receptor. (Nivolumab)	Compare efficacy and safety of nivolumab alone vs bevacizumab in recurrent GBM. Evaluate safety and tolerability of nivolumab alone and nivolumab + ipilimumab	Phase III 530 participants Randomized Parallel Assignment Open Label	Adverse events OS	Grade 3/4 treatment related adverse events were similar between groups. Median OS was 9.8 months (95% CI, 8.2–11.8 months) with nivolumab vs 10.0 months (95% CI, 9.0–11.8 months) with bevacizumab (HR, 1.04; 95% CI, 0.83–1.30; *p* = 0.76) PFS was 1.5 1.5 months (95% CI, 1.5–1.6 months) with nivolumab and 3.5 months (95% CI, 2.9–4.6 months) with bevacizumab (HR, 1.97; 95% CI, 1.57–2.48; *p* < 0.001)
NCT02617589 (Checkmate 498)	Nivolumab	Nivolumab + RT vs. RT + TMZ in MGMT unmethylated newly diagnosed GBM	Phase III 560 participants Randomized Parallel Assignment Open Label	OS	OS was 13.40 (12.62–14.29) in Nivolumab + RT vs. 14.88 (13.27 to 16.13)
NCT02667587 (Checkmate 548)	Nivolumab	Nivolumab + RT-TMZ vs. RT + TMZ in MGMT methylated newly diagnosed GBM	Phase III 716 participants Randomized Parallel Assignment Single-Blind	PFS per blinded independent central review (BICR) OS	No statistically significant improvement in PFS
NCT02336165	IgG1 monoclonal Ab against PD-L1 (Durvalumab—MEDI4736)	Durvalumab (MEDI4736) in newly diagnosed and recurrent glioblastoma (5 non comparative arms)	Phase II 159 participants Non-randomized Open Label	OS at 12 months PFS at 6 months OS at 6 months	Dur monotherapy appear to be well tolerated.

This table summarizes the most important research projects in Checkpoint Inhibitors Therapy.

**Table 4:** Chimeric Antigen Receptor (CAR) T Cell Therapy.

Clinical Trial	Chimeric Antigen Receptor	Description	Features	Primary Outcomes	Significant Results
NCT02209376	CART-EGFRvII Autologous T cells transduced with a lentiviral vector to express a CAR specific for EGFRvIII	Determine the safety and feasibility of CART-EGFRvII in the treatment of patients with EGFRvIII+ GBM with recurrence.	Phase I 11 participants Single Group Assignment Open Label	Adverse events	CART-EGFRvIII cells are safe. Active infiltration of activated CAR T cells, recruitment of new T cells.
NCT01454596	CART-EGFRvII	Evaluate safety and feasibility of administering T cells expressing CART-EGFRvIII to patients with malignant gliomas expressing EGFRvIII	Phase I/II 18 participants Non-randomized Sequential Assignment Open Label	Adverse events. PFS	Two patients experienced severe hypoxia, including one treatment related mortality after cell administration at the highest dose level. Median PFS was 1.3 months (interquartile range 1.1–1.9), with a single outlier of 12.5 months. Median OS was 6.9 months Two patients survived over one year, and 30% was alive at 59 months
NCT04003649	IL13-Rα2	Evaluate IL13-Rα2 Targeted CAR T Cells combined with CPI for patients with resectable recurrent GB	Phase 1 60 participants Randomized Parallel Assignment Open Label	Adverse events Dose-limiting toxicity (DLT) Feasibility OS	Estimated primary completion date: December 2022
NCT01109095	HER.CAR CMV-specific CTLs	Safe dose of HER2-CD28 CMV-T cells	Phase I 16 participants Single Group Assignment Open Label	Dose limiting toxicity	Safety of autologous HER2-CAR VSTs with no serious adverse events.

This table summarizes the most important research projects in CAR T Cell Therapy.

## Data Availability

Not applicable.
